# A Scoping Review of Pakistani Healthcare Simulation: Insights for Lower-Middle-Income Countries

**DOI:** 10.7759/cureus.76485

**Published:** 2024-12-27

**Authors:** Maria Bajwa, Fizza Najeeb, Haneen Alnazzawi, Ayesha Ayub, Jessica G Bell, Fouzia Sadiq

**Affiliations:** 1 Health Professions Education, Massachusetts General Hospital (MGH) Institute of Health Professions, Boston, USA; 2 The Center for Interprofessional Education and Practice (CIPEP), Nova Southeastern University, Fort Lauderdale, USA; 3 Internal Medicine, Shifa International Hospitals Limited, Islamabad, PAK; 4 Department of Surgery, Division of Anesthesiology, University of Jeddah, Jeddah, SAU; 5 Life Sciences, University of Management and Technology, Lahore, PAK; 6 Bellack Library, Massachusetts General Hospital (MGH) Institute of Health Professions, Boston, USA; 7 Research, Shifa Tameer-E-Millat University, Islamabad, PAK

**Keywords:** healthcare education, innovation, lmic, pakistan, scoping review, simulation

## Abstract

Healthcare simulation has gained global recognition in health professions education, yet its adoption in Pakistan, a lower-middle-income country (LMIC), remains limited. This scoping review aimed to explore how simulation is integrated into healthcare education in Pakistan, highlighting challenges and opportunities to inform similar LMICs. Pakistan serves as a critical case study for LMICs due to its unique challenges, including uneven access to simulation technologies and limited faculty training, which are shared by many similar resource-constrained settings. Using the Arksey and O’Malley framework, a systematic review of 693 publications identified 145 studies that met inclusion criteria. The findings revealed diverse simulation modalities primarily focused on skills training and clinical decision-making. Notable innovations included low-cost simulation solutions, effectively addressing resource constraints. However, significant gaps emerged, including an urban-centric focus with limited rural representation, insufficient evaluation of long-term impacts, and the absence of standardized terminology and training protocols. These challenges hinder broader integration and equitable access to simulation-based learning. Addressing these gaps through strategic collaborations, capacity-building initiatives, and innovative, cost-effective solutions, such as low-cost simulators crafted from readily available materials, could enhance simulation adoption in Pakistan and similar LMICs. This review highlights the importance of adopting evidence-based practices, increasing funding, and conducting comprehensive research on simulation's long-term impact to ensure effective implementation and improved healthcare education and outcomes globally.

## Introduction and background

Simulation-based education (SBE) in healthcare is an educational technique that replaces or amplifies real experiences with guided experiences that evoke or replicate substantial aspects of the real world in a fully interactive manner [1,2]. Over the years, SBE has emerged as a transformative approach for preparing healthcare professionals to manage the complexities of clinical practice [2]. SBE enables professionals to approach patient care confidently and competently by bridging the gap between theoretical knowledge and real-world application [2-4]. SBE provides a safe, controlled environment where learners can develop clinical skills, enhance decision-making, and foster teamwork and communication [2,5,6]. 

As an evolving field within healthcare education, SBE’s growth is reflected through the increasing number of publications and improvements in research quality [7,8]. Where the quantity of research shows this growth, the increasing rigor and sophistication of studies provide valuable insights into the depth of knowledge generated, the advancement of research methodologies, and the positive impact of simulation on healthcare outcomes [1,9,10]. While SBE has demonstrated its transformative impact globally, its adoption and implementation in lower-middle-income countries (LMICs) remain uneven, constrained by structural and resource limitations [2,11,12].

Home to nearly half of the world’s population, LMICs face significant barriers to fully integrating SBE, including resource limitations, inequitable access to training, and outdated educational methods, among several others [12]. With a projected healthcare workforce gap of 18 million by 2030, LMICs urgently need scalable, cost-effective, and capacity-building solutions to address disparities in healthcare education by providing standardized, experiential learning that enhances healthcare training across diverse settings [2,11].

As an LMIC, Pakistan exemplifies both the potential and challenges of SBE adoption, mirroring obstacles other LMICs face [2,11]. Pakistan’s healthcare education landscape, characterized by resource scarcity, regional disparities, and underdeveloped infrastructure, reflects the broader challenges LMICs face [2,11]. These challenges make Pakistan an optimal case study for understanding SBS's integration and its implications for similarly constrained settings [2,11]. Additionally, limited faculty training and uneven geographic access to resources create significant barriers to effective implementation [11]. These factors position Pakistan as an ideal case study to explore SBE's transformative potential in low-resource settings. Examining the integration of SBE into Pakistan’s education system can highlight critical gaps and opportunities, offering insights for other resource-constrained settings.

To our knowledge, no comprehensive review of SBE in Pakistan has been conducted. Scoping studies offer a structured approach to comprehensively exploring and mapping research literature, providing valuable insights into existing practices, identifying knowledge gaps, and providing a basis for a systematic review [13]. Using this methodology, our scoping review aims to evaluate the integration, adoption, and utilization of SBE methods, technologies, and research within healthcare education programs in Pakistan. This review seeks to identify areas for improvement and inform strategies by providing recommendations for improving the diffusion of simulation not only in Pakistan but also in other LMICs seeking to optimize SBE in resource-constrained environments.

## Review

Methods

Study Design

We conducted a scoping review according to Arksey and O’Malley (2005), refined by Levac et al. (2010), following the guidelines set forth by the Preferred Reporting Items for Systematic Reviews and Meta-Analysis with the associated extension for Scoping Reviews [13-16]. This framework facilitates the identification, mapping, and synthesis of a wide range of literature, which aligns well with the aims of our review, particularly in addressing the diverse and emerging nature of simulation-related studies [13]. Additionally, its flexible approach is well-suited to handle both published and gray literature, supporting the inclusivity necessary for a review of this scope [13]. The framework’s iterative and systematic process ensures that all relevant sources are considered, making it ideal for the expansive and evolving field of SBE in healthcare [13].

We followed the five steps of Arksey and O’Malley (2005), which included (1) developing research questions, (2) identifying relevant studies by establishing eligibility criteria, (3) selecting studies through database searches, (4) charting data using the online review management tool Covidence, and (5) collating, summarizing, and reporting the results [17,18]. The sixth step, an expert review, was considered fulfilled by having experts from simulation (MB and HA), research (FS and MB), and library science (JB) on the team [14].

(1) Developing the research questions: We framed our research questions using the Population (P), Concept (C), and Context (C) framework [19], as follows: 1. What is the current healthcare SBE and research status in Pakistan? 2. What factors influence SBE and research in Pakistan, as shown by the research studies? 3. As the studies revealed, what are the current gaps in healthcare simulation in the context of Pakistani healthcare education, and how can we fill these gaps?

(2) Identifying relevant studies: We used the following inclusion and exclusion criteria to identify studies.

Inclusion criteria: This included publications of any study designs that discussed SBE in healthcare, if: (1) the research was conducted in Pakistan, (2) the study was about healthcare education and training within the Pakistani healthcare system or community and public health using simulated teaching methods (any form of SBE), and (3) one or more of the researchers/authors worked in Pakistan and collaborated with counterpart outside of Pakistan for SBE-related research (a collaborative study with counterparts outside of Pakistan). We did not restrict our search to English as long as we had the article’s translation in English.

Exclusion criteria: We excluded studies if (1) research was conducted outside of Pakistan, (2) researchers did not work in Pakistan, even if of Pakistani origin, (3) the study was related to healthcare education but only suggested SBE without explicitly discussing or implementing it, as we aimed to include studies that provide concrete evidence of SBE’s application and impact, (4) the research was not related to healthcare, humans, or healthcare simulation, and (5) articles were not peer-reviewed.

(3) Search strategy and information sources: Following the eligibility criteria (Appendix A), our initial search in PubMed identified only 17 studies. However, being familiar with the healthcare education systems of both the United States and Pakistan, we realized that differences in terminology had caused the omission of relevant studies. We expanded our search using keywords from the identified studies to address this issue (Appendix A). We collaborated with a health sciences librarian, dedicating considerable time to refining and verifying the search strategy across PubMed, CINAHL, and PsycINFO (Appendix B), ensuring a more thorough and accurate search.

Recognizing the limited accessibility of international publication avenues for Pakistani researchers, we searched the local Pakistani database "PakMediNet," which was initially established to promote scholarship at the national level as a volunteer service by a group of physicians [20]. Given the challenges many Pakistani scholars face in publishing in international journals, this local database serves as an essential resource for capturing research outputs that might otherwise be overlooked. We also conducted manual searches of the included research studies. Using the same strategy, we conducted three database searches on September 25, 2023, February 29, 2024, and July 3, 2024, adding new studies to capture as many articles as possible before the final analysis. 

(4) Charting data:* *We chose Covidence as our review management tool because of its ability to streamline and make the process transparent during data charting [17,18]. We imported search results into Covidence, and by using Covidence’s step-by-step built-in approach, we conducted title and abstract screenings, full-text reviews, and data extraction within that platform. After obtaining inter-rater reliability (IRR) at every stage, two researchers from the entire team conducted the review and extracted data independently, while a third reviewer resolved any conflicts. Authors (MB and FS) constructed the data extraction form in Covidence after careful deliberation on answering the research questions and revised once the charting process started after conversations with other authors.

IRR: Initially, MB, FN, AA, and HA screened 10 titles and abstracts together to ensure consistent understanding within Covidence using the inclusion criteria. To achieve IRR, all researchers demonstrated the process in online meetings after the initial coaching session. Once IRR was established in the screening phase, any two reviewers independently screened titles and abstracts, ensuring that two authors reviewed each record. Any conflicts were resolved through discussions led by MB, supported by relevant literary evidence during weekly meetings. The IRR was measured using Covidence, with Cohen’s Kappa calculated at 0.81 for this phase (>0.6 value indicates a sufficient IRR) [17]. 

Robustness: Establishing the process of IRR was repeated during the full-text review and data extraction phases. Any two authors could extract the data, and a third author (MB) could resolve the conflict through an independent article review and mutual discussion. FN and HA overviewed all data extracted by MB. Throughout the charting process, MB, FN, AA, and HA took detailed notes, met weekly, and discussed the studies to minimize bias and maintain reflexivity, ensuring that personal biases did not influence the findings. The IRR was measured using Covidence, with Cohen’s Kappa calculated at 0.71 for this phase [17].

(5) Collating, summarizing, and reporting the data: After downloading the data from Covidence, MB and FN cleaned the data by reviewing each study and ensuring consistency in the information extracted. The two authors then collaboratively developed an initial coding framework based on the research questions and the key themes identified in the literature. The codes were generated inductively, drawing on the content of the studies, and were refined through iterative discussions between MB and FN to ensure alignment with the study’s objectives. To test the robustness of the coding framework, MB and FN independently coded a sample of studies, and any discrepancies were resolved through consensus. Following this, a thematic analysis focused on the publication foci, simulation content, outcomes, challenges, and facilitating factors was conducted. Themes were continuously revised and validated as the analysis progressed to ensure they accurately reflected the data and research questions. 

As this was a scoping review, statistical meta-analysis was not performed. However, the authors (MB and FN) conducted simple descriptive statistics for specific attributes, collected anecdotally and fact-checked against simulation literature. These attributes included healthcare professions, level of learners, level of intra- and inter-organization collaboration, collaboration within and outside of Pakistan, and study designs.

Since the terminology and the scope of healthcare professions differed somewhat between America and Pakistan, we established analysis guidelines through open discussions among all researchers. For example, when categorizing simulation studies discussing pediatric, gynecological, or otolaryngological fields involving medical students, we classified them under medical education, as this was the primary level of education being studied. Studies involving advanced learners, such as residents, fellows, and nurses in various specialties and subspecialties, were categorized as medicine (surgical or medical) or nursing, respectively (Table 4 in Results). Therefore, it was determined that the learner’s primary level should be considered first, followed by the content and context of the simulation.

Results

We organized the results according to the first two research questions for clarity in reporting. The third question was addressed through knowledge gained from the findings of the first two questions and is deliberated within the discussion section (Table 1).

**Table 1:** Outline of the results PRISMA - Preferred Reporting Items for Systematic Reviews and Meta-Analyses

Research Questions	Attributes	Sub-Attributes
1. Current status of simulation in Pakistan	Number of studies	Description of studies
		PRISMA flowchart
	Study aims/goals	One table
		No sub-attributes
	Demographic information of the articles	Publication year
		Level of collaborative work
		Mapping the distribution of collaboration
		Study design
		Healthcare professions
		Terminology used
		Funding
	Content of the articles	Topic of the articles
		Focus of the studies
		Reporting of sim sessions
		Using theories/frameworks as a guide
		Faculty training
		Simulation modalities
		Reporting of outcomes
2. Factors influencing simulation-based education and research in Pakistan	Simulation-related challenges	Resources associated
		Learner associated
		Simulation system associated
		Assessment associated
		Others
	Simulation-related facilitating factors	Resources associated
		Learner associated
		Simulation system associated
		Assessment associated
		Others
3. Gaps in healthcare simulation in the context of Pakistani healthcare education, and how can we fill these gaps	Knowledge synthesis occurred based on findings of the first two questions, addressed in the discussion section	Simulation in Pakistan: Innovation or not?
		Taxonomy and activities: Are we saying the same thing?
		Collaboration: How can we maximize the benefits?
		Simulation in healthcare education: When and how can we move beyond what we have?
		Simulation uses and content: What else can we do?
		Activities quality and reporting: Is this good enough?

Current status of simulation in Pakistan

Number of Studies

**Figure 1:**
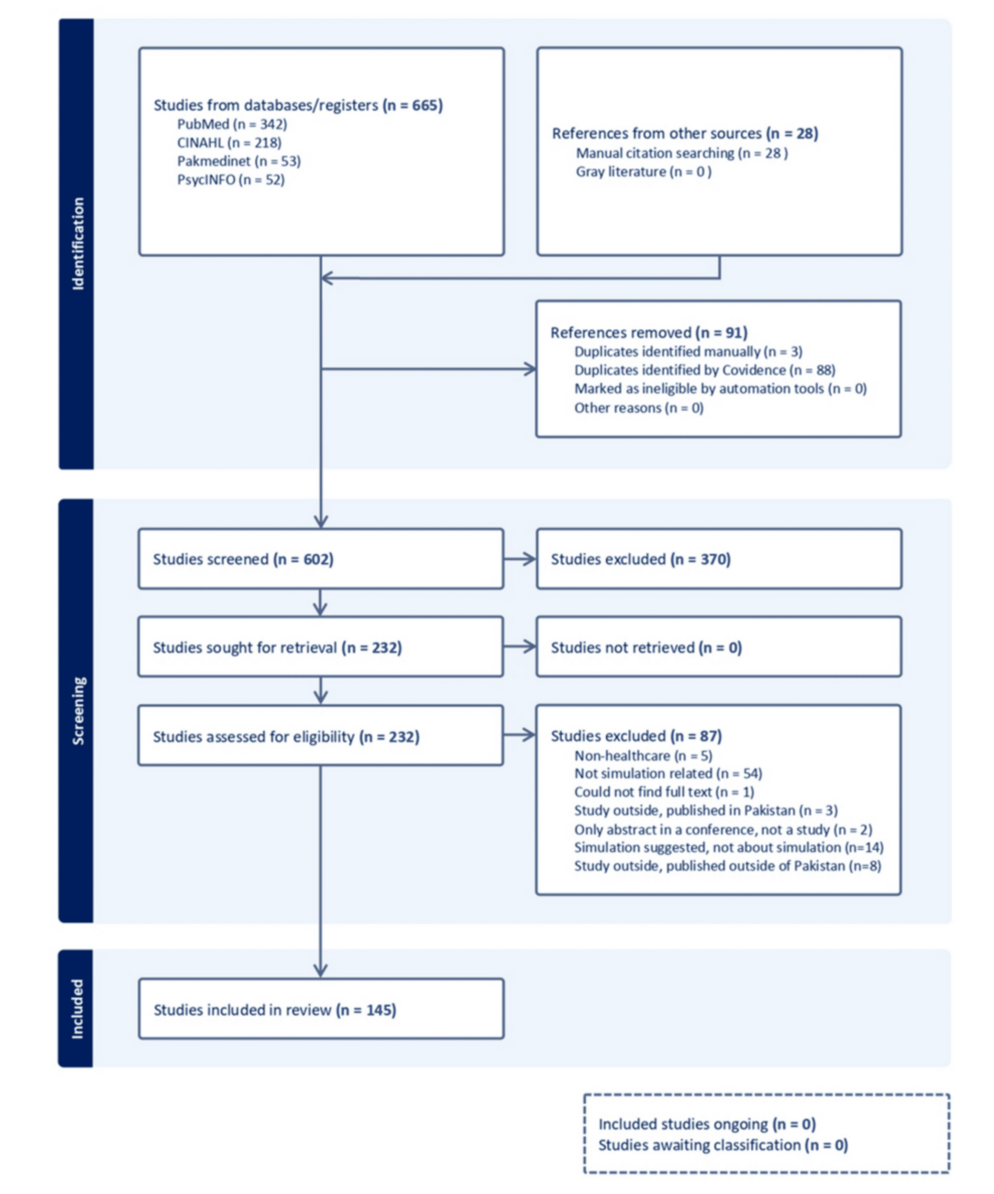
PRISMA flowchart for scoping review of Pakistan-based SBE research PRISMA - Preferred Reporting Items for Systematic Reviews and Meta-Analyses SBE - Simulation-based education

The three searches yielded 665 publications, which were uploaded to Covidence. This number increased to 693 after manually searching the references of the available articles. Data were extracted from the final body of knowledge, consisting of 145 studies (Figure 1).

Study Aim/Goal

The supplementary table in Appendix C lists the aims of the individual records and their references [21-165]. 

Demographic Information of the Articles

Under article demographics, we reported publication year, level of collaborative work, mapping the distribution of collaborative research, study design, healthcare professions, taxonomy/terminology, and funding. Interestingly, we did not find articles in any other language besides English and found no Urdu language journals or databases publishing or tracking healthcare simulation research in Urdu, the national language of Pakistan.

**Figure 2:**
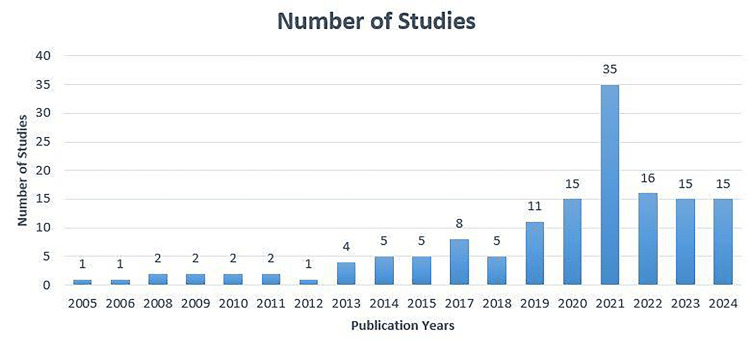
Distribution of simulation research studies published over the years

Publication year:** **In our dataset, the first study that described using simulation methodology was published in 2005, the only study in that year. The publication rate stayed under 10 studies per year until 2019, after which it trended upward with 15 studies in the first half of 2024, until July 3, when the last search was conducted (Figure 2). 

Level of collaborative work:The studies were divided into three collaboration levels (Table 2). We reviewed the details provided in the full-text articles to determine each author’s affiliation. The results were as follows: (1) Authors were in Pakistan; studies conducted and published in Pakistan (n=62, 42%), (2) authors were collaborating with authors abroad; studies conducted in Pakistan and published either within or outside of Pakistan (n=43, 29%: Pakistan [9], abroad [34]), and (3) authors were in Pakistan; studies conducted in Pakistan and published in journals outside Pakistan (abroad, foreign: n=40, 27%).

**Table 2:** Origin of studies according to the location of authors

Origin of studies (local or collaborative projects)	Publishing countries of journals	Studies Count (%) [numbers]
1) Local projects with Pakistani authors and publication in local Pakistani journals	Pakistan	62 (42%) [22, 24, 25, 29, 34, 45, 49, 51, 53, 54, 56, 57, 58, 59, 61, 62, 63, 64, 66, 71, 73, 74, 76, 77, 78, 80, 82, 83, 91, 93, 94, 95, 99, 101, 106, 107, 109, 110, 112, 113, 114, 116, 118, 119, 122, 126, 128, 129, 133, 136, 142, 144, 145, 151, 152, 153, 156, 157, 158, 159, 160, 165]
2) Collaborative projects (authors from both Pakistan and abroad) published locally and abroad	Total (Pakistan + abroad)	43 (29%)
	Abroad**	34 (23%)
		United Kingdom (13, 8%) [23, 27, 28, 42, 43, 85, 89, 100, 103, 104, 161, 163, 164]
		United States of America (9, 6%) [36, 52, 69, 102, 123, 124, 130, 139, 162]
		New Zealand (3, 2%) [72, 97, 115]
		Miscellaneous: Australia (2, 1%) [92,155], Switzerland (2, 1%) [39,140], Netherlands (1,0.6%)[31], Japan (1,0.6%) [40], Germany (1, 0.6%) [38], Spain (1, 0.6%) [65], Ireland (1, 0.6%) [150]
	Pakistan*	9 (6%) [21, 79, 90, 98, 132, 134, 137, 138, 143]
3) Local projects with Pakistani authors and publication in foreign journals	Abroad	40 (27%)
		United Kingdom (17, 11%) [26, 30, 35, 44, 46, 50, 68, 75, 86, 88, 96, 108, 120, 131, 141, 146, 148]
		United States of America (8, 5%) [41, 47, 48, 60, 84, 87, 117, 154]
		India (6, 4%) [32, 33, 55, 70, 105, 127]
		Miscellaneous: Japan (2, 1%) [147, 149], New Zealand (1, 0.6%) [37], Switzerland (1, 0.6%) [67], South Korea (1, 0.6%) [81], Scotland (1, 0.6%) [111], Turkey (1, 0.6%) [121], Iran (1, 0.6%) [125], Netherlands (1, 0.6%) [135]
*Denotes the studies that were conducted through collaborative efforts of Pakistani authors and foreign authors but were published in local journals of Pakistan. **Denotes the studies that were conducted through collaborative efforts of Pakistani authors and foreign authors but were published in foreign journals outside of Pakistan.		

**Figure 3:**
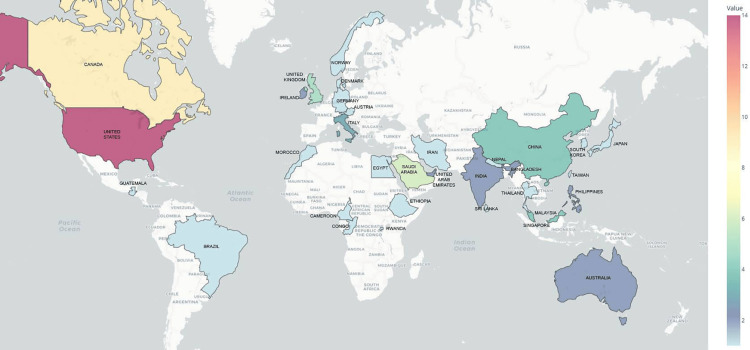
Global map indicating the collaborating countries and the number of studies

**Figure 4:**
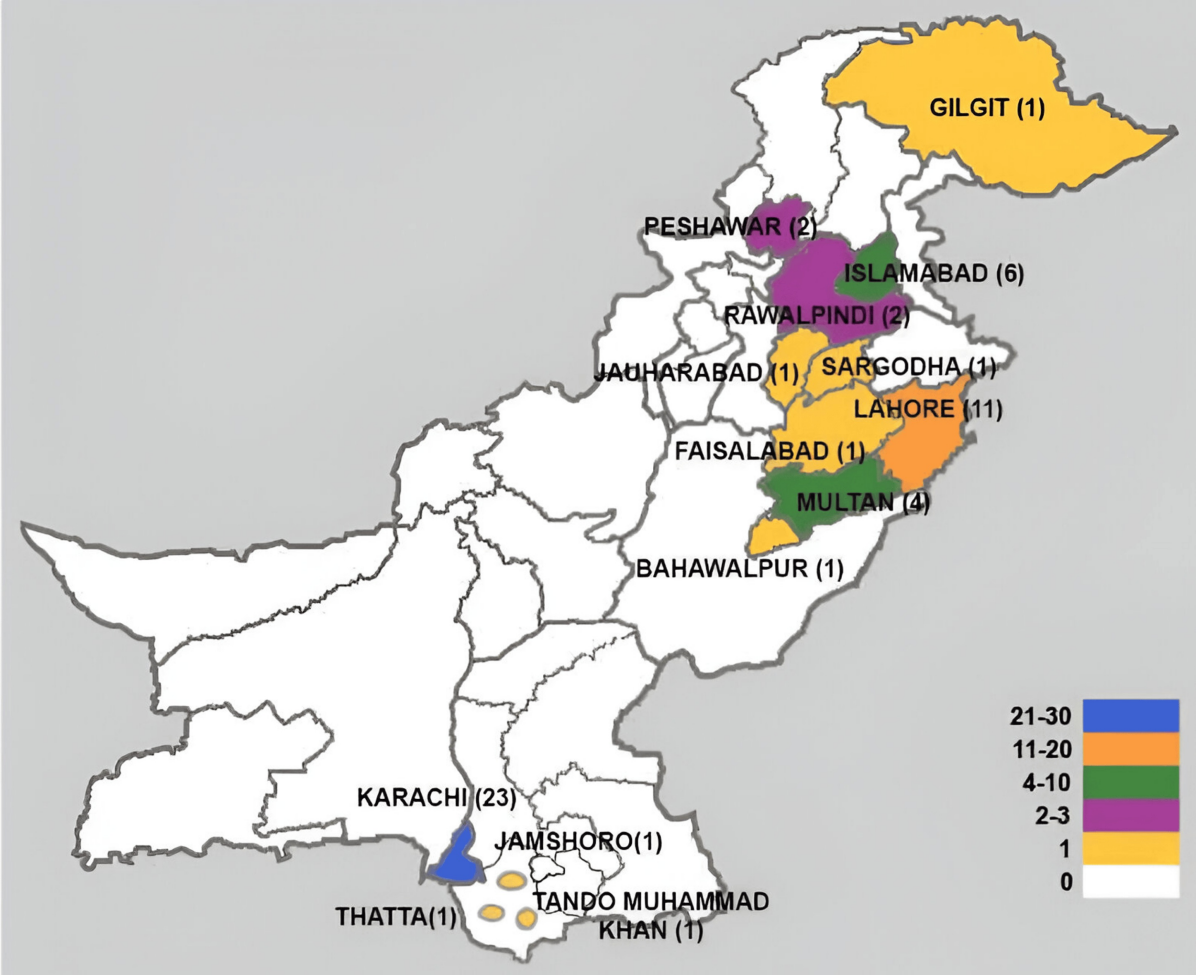
Cities of Pakistan involved in foreign collaborative simulation studies

**Figure 5:**
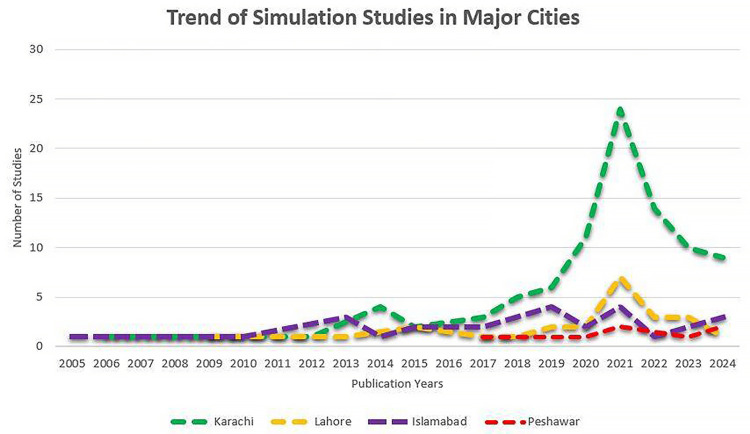
Trend of simulation studies conducted within bigger cities of Pakistan (federal or provincial capitals) through foreign collaboration

Mapping the distribution of collaboration: Seventeen studies were conducted through local collaborative efforts by authors from various major cities in Pakistan. These studies were primarily conducted in urban areas with relatively well-established medical and healthcare educational institutions. The participating cities included Karachi (n=10), Lahore (n=7), Islamabad (n=7), Peshawar (n=4), Rawalpindi (n=3), and one study each from Gujranwala, Bahawalpur, Quetta, Faisalabad, and Mardan. Forty-three studies were conducted through the collaborative efforts of authors from Pakistan and outside (Figure 3). The cities of Pakistan where the collaborative studies originated and their trend over time are illustrated in Figure 4 and Figure 5.

Study design: We found many study designs, and sometimes discrepancies were noted between the described and reported study designs. This discrepancy added complexity to the already diverse study collection. To simplify the vast array of studies, we divided them into (1) original research, (2) synthesis of knowledge, (3) professional communications, and (4) other (Table 3).

**Table 3:** Number of studies based on the study design

Study Design		Studies Count (%) [numbers]
Original Research (n=103)		
Quantitative studies (70)	Cross-sectional studies	30 (20%) [21, 28, 29, 35, 39, 40, 42, 44, 50, 54, 65, 66, 67, 70, 77, 80, 90, 92, 95, 97, 99, 102, 106, 117, 121, 122, 141, 142, 146, 160]
	Non-randomized experimental studies	22 (15%) [30, 33, 46, 47, 53, 59, 74, 79, 82, 85, 94, 98, 107, 108, 109, 126, 143, 147, 150, 152, 155, 164]
	Randomized control trials	11 (7%) [45, 88, 91, 96, 105, 118, 120, 132, 140, 145, 153]
	Quasi-experimental studies	7 (4%) [32, 41, 84, 114, 124, 137, 138]
Qualitative studies (2)	Interview studies	2 (1.3%) [51, 111]
	Focus group studies	1 (0.6%) [111]
Mixed method (9)	Questionnaire with focus group discussions*	2 (1.3%) [52,131]
	Deductive sequential mixed method study	1 (0.6%)[72]
	Observational mixed method study	1 (0.6%) [161]
	Triangulation mixed method study	1 (0.6%) [129]
	Explanatory sequential mixed method study	1 (0.6%)[56]
	Quantitative-qualitative mixed method study	1 (0.6%) [130]
	Quasi-experimental mixed method study	1 (0.6%) [25]
	Combined qualitative and quantitative mixed-method study	1 (0.6%) [73]
	*Did not name the mixed method design. Therefore, we are using their data-gathering method to classify the studies.	
Case reports	Case reports	22 (15%) [26, 31, 43, 60, 71, 75, 81, 86, 87, 93, 104, 113, 115, 119, 123, 125, 127, 128, 154, 156, 158, 162]
Synthesis of Knowledge (n=30)		
Review of literature	Narrative reviews	18 (12%) [34, 36, 37, 49, 57, 61, 63, 64, 83, 112, 133, 134, 136, 139, 144, 157, 163, 165]
	Systematic reviews	8 (5%) [23, 24, 27, 89, 116, 135, 148, 149]
	Literature reviews	3 (2%) [22, 55, 76]
	Scoping reviews	1 (0.6%) [69]
Professional Communication (n=11)		
Narrative/ communicative articles	- Opinion	4 (2.7%) [68, 100, 110, 159]
	Short communication	3 (2%) [48, 58, 151]
	Letter to editor	2 (1.3%) [38, 101]
	Editorial	1 (0.6%) [78]
	Innovation	1 (0.6%) [62]
Other: Pre-Research Planning (n=1)		
Study protocol	Study protocol	1 (0.6%) [103]

Healthcare professions: We did not exclude any healthcare professions that reported using simulation. The five most common professions using simulation techniques were medicine (100), followed by studies involving multiple professions (11), community and public health (10), nursing (9), and dentistry (7). For ease of description, we included surgery, medical education, and medicine within the medical field, which might differ in other countries [84,98,158].

A few studies involved more than one healthcare profession. Studies were categorized as interprofessional education (IPE) if they reported participants working together as a team, aligning with the WHO’s definition of IPE: learningfrom, with, and about each other. If participants did not work together in this manner, as the WHO intended, the study was categorized as multiprofessional education (Table 4). 

**Table 4:** Healthcare professions reported in the data set

Professions Involved	Description	Studies Count (%) [numbers]
Medical	Total (surgery, medicine, and medical education combined)	101 (69%)
	Surgery	45 (31%) [22, 23, 24, 31, 34, 36, 38, 44, 54, 62, 63, 64, 67, 68, 71, 75, 78, 83, 84, 86, 91, 92, 102, 107, 110, 112, 113, 116, 121, 123, 125, 126, 133, 136, 139, 144, 145, 147, 148, 149, 152, 157, 159, 162, 165]
	Medical education	36 (24%) [29, 32, 33, 35, 37, 42, 47, 53, 70, 73, 74, 77, 80, 94, 95, 98, 100, 101, 105, 106, 119, 120, 122, 127, 129, 131, 134, 137, 138, 141, 142, 143, 151, 153, 156, 163]
	Medicine	20 (13%) [30, 41, 43, 45, 49, 52, 56, 59, 60, 66, 72, 89, 90, 96, 103, 128, 135, 146, 155, 158]
Multiprofessional/interprofessional education (IPE)	Total (multiprofessional and IPE)	11 (7%) (medicine, nursing, dentistry, surgery) [27, 28, 48, 51, 79, 81, 115, 130, 150, 160, 161]
	More than one profession reported learning with, from, and about each other (interprofessional simulation)	3 (2%) [79, 81, 130]
	Not reported as IPE (multiprofessional simulation)	8 (5%) [27, 28, 48, 51, 115, 150, 160, 161]
Community/public health	-	10 (6.8%) [25, 82, 85, 93, 104, 108, 109, 111, 114, 117]
Nursing	Total (nursing profession only)	9 (6.2%)
	Nursing students	7 (4.8%) [26, 50, 58, 69, 88, 124, 154]
	Working nursing professionals	2 (1.3%) [46, 87]
Dentistry	-	7 (4.8%) [55, 57, 61, 76, 99, 132, 164]
Pharmacy	-	6 (4.3%) [39, 40, 65, 97, 118, 140]
Others	Paramedics	1 (0.6%) [21]

Terminology used:** **An internationally recognized healthcare simulation dictionary, freely accessible [166], guided this scoping review, providing standardized terminology for simulation. Drawing on professional experiences in two countries outside the United States, MB and HA observed regional variations in simulation taxonomy and terminology. This disparity became evident during the initial search (Appendix A), which identified only 19 articles. These geographical differences in simulation methodologies underscored the need to address the knowledge gap between Pakistan and the global community.

In our search, “simulation” was the most common term used to describe simulated methodologies for healthcare education; however, the first study in our dataset used the words “train to act” to describe simulated participant (SP), an established simulation methodology [66,166]. Other terms included “training,” “skills training,” “resuscitation,” “basic life support,” “wet lab training,” “OSCE,” “simulated patient,” “simulated client (SC),” and “role-play.” These alternative terms were often not used alongside the term "simulation."

Funding: Only 26 (17%) studies reported receiving funding, while 119 did not provide any funding information [25,26,28,33,42,52,62,65,85-87,97,101-103,106,108,111,115,117,124,130,131,140,150,161]. Apart from collaborative projects with international funding, most of the financial support for local projects was provided by the institutions where the research was conducted. Few studies defined their funding sources and declared that their findings were not influenced or biased by their funding bodies [47,123,150,158,161]. 

Content of the Articles

To better understand the landscape of simulation education and research, we categorized the content presented in the dataset into the following themes: the topic of the articles, study focus, reporting of simulation sessions, using theories/frameworks as a guide, faculty training, simulation modalities, reporting of outcomes, and challenges and facilitating factors.

Topic of the articles: These 145 articles discussed simulation in various ways, such as an intervention, a topic of their review of the literature, or professional communications. The topics included studies that discussed surgical skills (35), some reported establishing the significance of simulation as an instructional method (30), and some focused on procedural skills (Table 5) [17].

**Table 5:** Studies based on the topic and focus of articles

The Topic of the Articles	
Attributes	Studies Count (%) [numbers]
Surgery/surgical skills	35 (24%) [23, 24, 31, 33, 36, 44, 62, 64, 67, 71, 75, 78, 83, 84, 86, 91, 92, 102, 112, 113, 116, 121, 123, 125, 126, 133, 136, 138, 144, 145, 147, 148, 149, 152, 165]
Establishing the value of simulation as an instructional method	30 (20%) [26, 29, 34, 37, 42, 43, 45, 50, 56, 61, 70, 74, 77, 80, 81, 89, 95, 96, 100, 101, 105, 106, 115, 122, 127, 134, 146, 151, 154, 157]
Procedural skills	17 (11%) [28, 30, 32, 35, 41, 59, 69, 72, 90, 93, 94, 103, 135, 143, 155, 161, 164]
Advanced technologies (three-dimensional printing, augmented reality, virtual reality)	17 (11%) [22, 38, 47, 49, 54, 55, 57, 63, 76, 99, 107, 110, 137, 139, 159, 162, 163]
Resuscitation/life support	16 (11%) [21, 25, 27, 53, 60, 82, 85, 88, 104, 108, 109, 111, 114, 132, 150, 160]
Physical exam/clinical skills/management-focused studies	11 (7%) [46, 58, 66, 79, 98, 124, 128, 129, 131, 141, 153]
Soft skills (communication, breaking bad news, etc.)	8 (5%) [51, 52, 73, 87, 120, 130, 156, 158]
Pharmacy services	7 (4%) [39, 40, 65, 97, 117, 118, 140]
Simulation center related	4 (2%) [48, 68, 119, 142]
Focus of Studies	
Discussion about simulation as a learning tool	37 (25%) [22, 23, 24, 27, 34, 36, 37, 38, 49, 55, 57, 61, 63, 64, 69, 76, 78, 83, 89, 100, 101, 110, 112, 116, 133, 134, 135, 136, 139, 144, 148, 149, 151, 157, 159, 163, 165]
Simulation commodity and assets (tool/framework, simulation center, simulator, task trainer)	31 (21%) [31, 41, 48, 62, 67, 68, 71, 72, 75, 80, 86, 87, 88, 90, 91, 92, 93, 99, 103, 113, 119, 121, 123, 125, 127, 128, 137, 138, 142, 147, 162]
Professional development	28 (19%) [21, 28, 30, 42, 43, 44, 46, 51, 52, 54, 56, 59, 60, 77, 81, 84, 102, 107, 108, 111, 115, 126, 130, 146, 150, 152, 155, 161]
Teaching content to students	20 (13%) [26, 32, 35, 47, 50, 53, 58, 73, 105, 106, 120, 122, 124, 129, 132, 140, 141, 143, 145, 154]
Assessment of simulation technique as an instructional method	15 (10%) [29, 33, 45, 70, 74, 79, 94, 95, 96, 98, 131, 153, 156, 158, 164]
Healthcare practice assessment	8 (5%) [39, 40, 65, 66, 97, 117, 118, 160]
Community outreach	6 (4%) [25, 82, 85, 104, 109, 114]

Study focus: We gathered data about how simulation was used in this body of knowledge, whether for teaching students, faculty development, teaching healthcare content using simulation, etc. Several studies explored multiple aspects of simulation; however, we categorized each study based on its primary focus. For example, Liaqat et al. primarily reported using simulation to assess the pedagogical framework for neonatal resuscitation and teaching content to learners [88]. Therefore, we categorized it under simulation commodity as the focus of study, the theme that deals with frameworks and physical resources, and as resuscitation/life support as the topic of the articles. Most common themes included discussions about the use of simulation as a learning tool (37, 25%), simulation’s commodities or assets (developing/validating theoretical tools/frameworks, simulator, task trainer, or simulation center; 31, 21%), and professional development (28, 19%) (Table 5).

Reporting of simulation session: This category encompassed various study designs, with 103 articles (71%) reporting simulation sessions at different levels of detail. Nearly all of these articles described simulation activities (100), pre-briefing (33), and debriefing (32). While every article included one or more assessment forms, several lacked essential details. 

Using a framework or theory as a guide: Most studies (82, 57%) did not reference any underlying theory or framework guiding their research, while a substantial portion (63, 43%) reported using theoretical frameworks (Table 6).

**Table 6:** Theoretical frameworks used in the dataset ADDIE - Analysis, Design, Development, Implementation, Evaluation

Theories/Framework	Studies Count (%) [numbers]
Others	30 (21%) [30, 41, 43, 44, 51, 52, 56, 58, 60, 66, 72, 73, 74, 77, 81, 86, 87, 93, 102, 104, 113, 120, 121, 127, 137, 138, 141, 151, 154, 162]
Preferred reporting items for systematic reviews and meta-analysis	9 (6%) [23, 24, 27, 69, 89, 116, 135, 148, 149]
American Heart Association guidelines	8 (5%) [21, 25, 53, 82, 85, 109, 132, 160]
Kolb’s experiential learning theory	3 (2%) [50, 84, 112]
Kirkpatrick evaluation model	3 (2%) [59, 130, 148]
Helping babies breathe/neonatal resuscitation program	3 (2%) [108, 111, 150]
"See, practice, do" framework	2 (1.3%) [88, 131]
Population/problem, intervention exposure, comparison, outcome	2 (1.3%) [27, 89]
McGill Inanimate System for Training and Evaluation of Laparoscopic Skills	2(1.3%) [91, 92]
"Plan-do-study-act" model	2 (1.3%) [46, 164]
ADDIE model	2 (1.3%) [128, 163]
Simulated client method	1(0.6%) [39]
Note: While not all studies employed a theoretical framework to guide their research or reporting, several studies used more than one framework. As a result, the reported numbers do not represent the total number of studies.	

Faculty training: In this body of knowledge, 54 (52%) studies reported training or professional development for faculty or staff involved in simulation-related activities. More than half of the studies (91, 48%) did not discuss the professional development of simulation users. Notably, one study did not report its own faculty training to conduct simulation but advocated for faculty training [77].

Simulation modalities: Different modalities were discussed depending on the type of the study article. Nine studies used simulation without mentioning the modality. Additionally, the concept of modality did not apply to 32 studies because some discussed an idea related to simulation and not a modality within the literature review or professional communication. The most commonly reported modalities per standardized definitions included virtual simulation (25, 17%), followed by SP (22, 15%), and manikin-based simulation (19, 13%) (Table 7) [166]. Since most of the studies were conducted in or around the cities of federal and provincial capitals, we did not find any studies conducted in the rural areas.

**Table 7:** Number of studies using various types of simulation modalities Simulation modalities are terms used to refer to the type(s) of simulation being used as part of the simulation activity, for example, task trainers, manikin based, etc. [166]. 3D - Three-dimensional

^Simulation Modality^	Studies Count (%) [numbers]
^Not applicable^	32 (22%) [22, 23, 24, 27, 34, 36, 37, 48, 49, 51, 61, 64, 69, 77, 78, 83, 89, 100, 101, 112, 113, 116, 133, 134, 135, 136, 144, 148, 149, 151, 157, 165]
Virtual simulation	24 (16%) [33, 38, 41, 47, 52, 54, 57, 62, 76, 86, 99, 102, 107, 110, 121, 123, 128, 137, 138, 139, 145, 152, 159, 163]
Standardized patients	22 (15%) [29, 39, 40, 42, 43, 50, 58, 65, 66, 70, 73, 74, 80, 87, 94, 97, 117, 118, 120, 124, 156, 158]
Manikins	19 (13%) [21, 25, 53, 59, 82, 85, 88, 98, 104, 105, 108, 115, 127, 129, 131, 132, 140, 142, 143]
Hybrid (more than one modality)	15 (10%) [26, 28, 30, 32, 45, 46, 72, 79, 81, 96, 106, 119, 141, 153, 154]
Models/simulators (in-house/innovation)	11 (7%) [31, 67, 84, 91, 92, 93, 103, 125, 147, 150, 164]
Not reported	9 (6%) [44, 56, 60, 111, 114, 122, 130, 146, 160]
Animal cadavers	5 (3%) [68, 71, 75, 90, 126]
Models (commercial)	4 (2%) [35, 109, 155, 161]
3D printing	3 (2%) [55, 63, 162]
Others	1 (0.6%) [95]

Reporting of outcomes: Studies reported their outcomes in different ways. To gauge the outcome more uniformly, we used the Kirkpatrick model of outcome evaluation for simulation-based intervention studies [103,167]. Some studies suggested behavioral changes (level 3) in their outcomes but lacked sufficient evidence to confirm them, so we categorized them as level 2 [114]. We did not evaluate publications related to the review of the literature (30), professional communications (11), and a study protocol because we found the Kirkpatrick model to be not genuinely applicable to these studies (Table 8).

**Table 8:** Studies evaluated using the levels of KP's model KP - Kirkpatrick

Outcome Status	Details	Description	Studies Count (%) [numbers]
KP’s levels applied for sim-based interventions^167^	KP level 2	The degree to which participants acquire the intended knowledge, skills, attitude, confidence, and commitment based on their participation in the training	68 (47%) [21, 25, 26, 28, 29, 30, 31, 32, 33, 39, 40, 41, 44, 45, 46, 47, 50, 52, 53, 54, 56, 59, 60, 65, 66, 68, 71, 72, 74, 82, 88, 91, 94, 95, 96, 98, 104, 105, 107, 108, 114, 117, 118, 120, 121, 123, 124, 125, 126, 129, 130, 131, 132, 140, 142, 143, 145, 147, 150, 152, 153, 154, 155, 156, 158, 160, 161, 164]
				KP level 1	The degree to which participants find the training favorable, engaging, and relevant to their jobs	23 (16%) [35, 51, 58, 70, 73, 80, 81, 87, 90, 92, 93, 99, 102, 106, 115, 119, 122, 127, 128, 137, 138, 141, 146]
	KP level 3	The degree to which participants apply what they learned during training when they are back on the job	6 (4%) [43, 79, 84, 85, 109, 111]
	KP level 4	The degree to which targeted outcomes occur as a result of the training and the support and accountability package	1* (0.6%) [111]
	Not applicable	-	Not applied: reviews, professional communication	39 (27%) [22, 23, 24, 27, 34, 36, 37, 38, 48, 49, 55, 57, 61, 62, 63, 64, 69, 76, 78, 83, 89, 100, 101, 110, 112, 116, 133, 134, 135, 136, 139, 144, 148, 149, 151, 157, 159, 163, 165]
Not reported	-	No outcomes reported	6 (4%) [75, 77, 86, 103, 113, 162]
Measured otherwise	-	Description of a simulator	3 (2%) [42, 67, 97]
*Study with the potential to be at level 4 with clearer reporting [111]			

Factors influencing SBE and research in Pakistan****

Simulation-Related Challenges

In this body of knowledge, 92 (63%) reported barriers in the simulation field, while 53 (36.5%) studies did not mention such obstacles. Key challenges in implementing SBE included limited resources (30 studies), inadequate infrastructure (20 studies), and a shortage of trained experts (6 studies), all of which limited the effectiveness of simulation in healthcare education (Table 9).

**Table 9:** Simulation-specific barriers reported by authors of our dataset

Simulation-Related Barriers/Challenges	Studies Count (%) [numbers]
Resources Associated	
Lack of funds for acquisition and maintenance	30 (21%) [22, 23, 24, 25, 34, 43, 44, 55, 63, 64, 70, 76, 77, 79, 99, 102, 103, 107, 112, 114, 117, 119, 135, 137, 143, 145, 146, 151, 161, 165]
Lack of appropriate equipment and infrastructure	20 (14%) [21, 22, 26, 32, 34, 35, 43, 61, 67, 70, 75, 77, 79, 104, 105, 112, 119, 141, 143, 146]
Short duration for training	14 (9%) [21, 28, 29, 52, 79, 85, 105, 114, 115, 129, 137, 145, 150, 164]
Inadequate planning time	8 (5%) [21, 32, 55, 70, 77, 87, 94, 164]
Lack of appropriately trained experts	6 (4%) [22, 79, 93, 122, 140, 151]
Lack of relevant simulation curricula (appropriate scenarios and consistent outcomes)	4 (2.7%) [43, 44, 52, 147]
Learner Associated	
Small sample size and lack of participation in follow-up studies	16 (11%) [30, 33, 80, 84, 85, 104, 106, 108, 109, 116, 140, 143, 145, 147, 148, 161]
Variability in performance due to previous exposure to simulation	5 (3%) [28, 44, 121, 135, 140]
Lack of simulation awareness/buy-in	5 (3%) [25, 35, 56, 99, 109]
Language barrier	2 (1.3%) [21, 47]
Simulation System Associated	
Lack of realism in:	13 (8%)
A- Scenario design	5 (3%) [27, 88, 134, 135, 143]
B- Models/manikins	3 (2%) [63, 109, 155]
C- Virtual simulation	3(2%) [47, 123, 145]
D- Task trainers	2 (1.3%) [67, 79]
Lack of force feedback/haptic feedback	3 (2%) [22, 47, 86]
Limited software capability	2 (1.3%) [63, 123]
Assessment Associated	
Lack of assessment of skill retention and long-term impact	12 (8%) [33, 41, 80, 88, 98, 102, 109, 115, 126, 134, 150, 155]
Reduced inter-rater reliability	12 (8%) [27, 39, 40, 44, 52, 65, 85, 94, 98, 106, 127, 153]
Performance variability of standardized patients/simulated clients	8 (5%) [26, 39, 40, 73, 87, 97, 140, 153]
Suboptimal study design and/or use of a framework	5 (3%) [69, 84, 148, 149, 161]
Others	
Such as incomplete literature review, unavailability of recent literature, publication bias, lack of generalizability	13 (8%) [23, 30, 51, 59, 60, 73, 89, 111, 116, 120, 134, 158, 163]
Note: A lack of uniformity was observed when reporting the modalities. We used the Healthcare Simulation Dictionary to guide us in this process.	

Simulation-Related Facilitating Factors

In this body of knowledge, 84 studies (57.9%) reported facilitating factors compared to 61 (42%) that did not. Key facilitating factors for SBE included cost-effective modalities (23) and abundant opportunities for experiential learning (33). Additionally, the use of validated assessment tools (11) and high-fidelity simulation scenarios (11) contributed to the effectiveness of simulation in healthcare education. These factors enhance the accessibility, quality, and impact of simulation in various healthcare settings (Table 10).

**Table 10:** Simulation-specific facilitating factors reported in our dataset

Simulation-Related Facilitating Factors	Studies Count (%) [numbers]
Resources Associated	
Cost-effective modalities	23 (16%) [23, 32, 43, 44, 45, 52, 67, 70, 71, 75, 91, 92, 103, 104, 108, 114, 123, 126, 127, 140, 147, 155, 161]
Appropriately trained educators	8 (5%) [69, 70, 72, 87, 108, 109, 128, 129]
Accessibility to relevant simulation curricula (appropriate scenarios and consistent outcomes)	4 (2.7%) [28, 43, 120, 128]
Appropriate equipment and infrastructure	3 (2%) [50, 68, 150]
Appropriately trained standardized patients	2 (1.3%) [66, 97]
Reduced cost by simulator sharing	1 (0.6%) [23]
Learner Associated	
Improved comprehensibility of content through translation into local languages	1 (0.6%) [25]
Positive participant attitudes due to pre-intervention counseling	1 (0.6%) [82]
Simulation System Associated	
Abundant opportunities for experiential learning	33 (23%) [27, 28, 33, 37, 45, 50, 56, 64, 79, 84, 88, 90, 91, 106, 126, 127, 129, 131, 135, 137, 139, 143, 144, 146, 152, 154, 155, 159, 161, 162, 163, 164, 165]
High-fidelity simulation scenarios	11 (7%) [26, 28, 47, 54, 87, 90, 93, 104, 140, 155, 163]
Portable, updatable, and open-access simulators	4 (2.7%) [86, 102, 155, 161]
User-friendly interface	1 (0.6%) [123]
Use of Evidence-Based Practices	
Validated means of assessment	11 (7%) [30, 32, 40, 67, 77, 85, 88, 91, 124, 148, 163]
Immediate debrief to improve learning	4 (2.7%) [28, 50, 94, 129]
Use of theoretical frameworks	3 (2%) [24, 154, 164]
Others	
Such as: Use of software tools for managing studies, use of validated metrics for simulator assessment, improvement of human performance, generalizability of results, real-life observation of procedures	12 (8%) [22, 24, 27, 39, 89, 111, 112, 117, 141, 152, 153, 162]

Discussion****

This scoping review examined the current state of SBE and research in Pakistan and the factors influencing its adoption. Findings revealed key gaps and potential solutions, which we discussed by integrating the results. This review moves the field forward by identifying challenges in Pakistan’s resource-constrained settings, thus providing opportunities to enhance SBE adoption and effectiveness. In this section, we discuss the challenges and their proposed solutions.

Simulation in Pakistan: Innovation or Not?

“An innovation is an idea, practice, or project perceived as new by an individual or other unit of adoption” [168]. This concept applies to SBE in LMICs such as Pakistan. Our scoping review revealed a steady increase in SBE-related publications over the years, with a sharp rise in the past five years. This relatively sudden increase in simulation studies contrasts with the decades-long adoption of SBE in well-resourced countries [1,12]. This increase also indicates growing social acceptance and local adoption among healthcare educators and professionals in Pakistan. Therefore, simulation methodology can be viewed as an innovation within Pakistani healthcare education, as it is a relatively new practice gaining traction and contributing to the development of healthcare training.

A key challenge in LMICs is the limited access to and maintenance of high-tech simulation models and mannequins, commonly available in resource-rich settings. This constraint has spurred innovative, cost-effective solutions, such as homemade laparoscopic endotrainers, repurposed disposable gloves, recorded clinical encounters for training, and validated rating scales [92,95,125,147]. These examples highlight how resourceful educators equipped with simulation knowledge can overcome financial and infrastructural barriers to gradually integrate simulation into Pakistan’s healthcare education system. These instances exemplify the need for institutional support to scale such innovations and for educators to share best practices through local and international collaborations to maximize impact.

Additionally, as revealed (Table 9), these innovative uses of simulation, coupled with positive learner experiences, became an advocacy point, reduced user hesitancy, and increased motivation and learning [25,35,99]. The lessons learned from similar activities also provided insights for educators and researchers for future iterations [24,154]. Overall, these factors combined are likely to promote greater acceptance of simulation among key stakeholders, including students, faculty, staff, administration, and the general public.

Since 2014, virtual simulation in Pakistan has evolved, with a fluctuating focus on assessing perceptions and teaching applications in fields such as radiology, urology, and ophthalmology [30,46,96,99]. Virtual or digital simulation has proven to be a promising tool for surgical training in LMICs and should thus be explored further [2,146]. The shift from perception to knowledge assessment to real-life skill application reflects the growing maturity of SBE [30,81,84].

Our review found no studies on integrating artificial intelligence (AI) into simulation. The lack of simulations using AI could be because it has been recently democratized and made accessible for everyone. We prompt healthcare simulationists to leverage AI in simulation to fill this crucial gap, as AI is a potentially revolutionizing tool for healthcare learning outcomes through simulation [169].

Taxonomy and Activities: Are We Saying the Same Thing?

A key finding is the inconsistent use of simulation-related terminology, reflecting the diverse views on what constitutes simulation in healthcare (see Terminology in Results). This variation creates barriers to sharing knowledge and evidence-based practices internationally. For instance, some studies used broad terms such as “integrated practical examinations” for simulation [141]. Similarly, the objective structured clinical examination (OSCE) was generally framed as an assessment method and an umbrella term for simulation and non-simulation activities [70,141]. The OSCEs sometimes consisted of multiple-choice questions and were later modified at one institution using SP [70]. A few studies used the words “training” or “resuscitation” and did not mention simulation, simulation phases, or its specific taxonomy in the reporting. 

The lack of clarity on what constitutes evidence-based simulation practices may stem from the lack of knowledge and training about simulation and the overuse of “simulation” as a buzzword [1]. This inconsistency hinders comparisons across studies and highlights the need for a standardized taxonomy aligned with international healthcare simulation standards [1]. One way to address this could be using the simulation dictionary and evidence-based practices to standardize simulation practices, conduct simulation-based research, and report related activities [1,166]. Efforts to align local simulation practices with international frameworks, such as adopting a standardized taxonomy, could ensure clarity and foster collaboration across borders.

Collaboration: How Can We Maximize the Benefits?

Local and international collaboration with high- and other low-resource settings is foundational in advancing SBE, especially in resource-limited settings such as Pakistan [11,12]. As observed, sharing resources and expertise helped alleviate financial constraints [11,24,37,144]. Designing self-sustaining simulation programs tailored to local needs and establishing simulation centers offering standardized training can provide cost-effective solutions, ensure consistent training, and reduce the need for clinicians to seek training abroad [26,37,144]. These centers can also incorporate cultural considerations, making the training more relevant to the local healthcare environment, a concern evident in collaborations between LMIC and high-income countries and regions [12,26]. Partnerships involving international funding and expertise have successfully overcome resource limitations and established sustainable simulation programs, thus promoting the concept [36,102]. 

Reporting SBE in the local context is essential to its broader adoption and in preparing healthcare professionals to meet local patient needs, considering various cultural aspects. Our research revealed a noticeable lack of simulation-related publications in local healthcare journals compared to other healthcare topics, a global gap evident by only a few SBE journals. While researchers aim to publish in prestigious journals, limited resources may restrict their ability to do so, reducing the visibility of local research. This could be minimized through formal agreements with national and international journals and by fostering knowledge sharing among regional institutions. 

Most studies have been conducted in Pakistan’s major cities, limiting their applicability to rural areas with fewer resources. This suggests the need for more SBE research in underserved regions and raises the possibility that smaller institutions may conduct SBE but lack the resources to publish. Programs in smaller cities can collaborate with more resourced programs to overcome these hurdles. This finding leads to two conclusions: research from low-income countries can inform practices in other underdeveloped regions, and many simulation activities may remain unreported due to resource constraints. Policymakers and institutional leaders should prioritize creating funding mechanisms and knowledge-sharing platforms to enable equitable simulation access across all regions.

Simulation in Healthcare Education: When and How Can We Move Beyond What We Have?

We observed a considerable focus among learners and educators on assessing perceptions of simulation [99,115]. While evaluating perceptions highlights the importance of simulation and enhances motivation, the focus must shift toward measuring actual outcomes of SBE, particularly in skill acquisition and clinical competence. SBE demonstrates lasting benefits, including long-term retention of knowledge and skills, successful skill transfer to clinical practice, and enhanced competence and confidence among participants, as seen in CPR, laparoscopic, and helping babies breathe (HBB) training programs. Participants also reported positive experiences, fostering acceptance and advocacy for SBE, which supports its broader adoption as an effective healthcare training modality. Where possible, priority should be given to long-term outcome evaluations to assess skill retention and behavioral changes rather than just short-term achievements [102,115,126].

Moreover, several faculty members involved in simulation were not trained explicitly in simulation techniques but were content experts [115,155]. Lack of user training for simulation can potentially limit the effectiveness of simulation activities and increase the cognitive load of both educators and learners [1,170]. Consequently, leveraging the faculty’s existing skill set can augment simulation integration [70,128]. This practice will broaden the faculty’s understanding of simulation’s potential beyond its traditional use in OSCEs and CPR training, enabling its application across diverse scenarios in healthcare education. For professional development, lack of funding was reported to be a barrier (Table 9). The need to have dedicated funding and leadership support for professional development initiatives may solve some of the issues mentioned above. Faculty development programs and longitudinal studies assessing skill retention and clinical competence could provide the foundation for sustainable SBE adoption in LMICs.

A notable finding was the relative absence of articles by Pakistani authors or published in Pakistan within the systematic reviews [23,24,27,89,116,135,148,149]. Possible reasons include a lack of studies in this area, poor reporting leading to exclusion due to high bias risk, or limited publication opportunities in peer-reviewed journals, often due to funding constraints. Further research is needed to investigate these issues.

We noted a significant lack of focus on IPE in this body of work. Only 11 studies involved more than one health profession, and few explicitly embraced the true definition of IPE, where two or more professions learn with, from, and about each other [171]. As a result, we categorized these studies as multiprofessional rather than interprofessional. IPE fosters teamwork, collaboration, role understanding, and improved service delivery, enabling learners to achieve outcomes only possible through multidisciplinary engagement [171].

Simulation Uses and Content: What Else Can We Do?

Simulation use varies across regions and healthcare professions in Pakistan, with the medical profession reporting the most. It is encouraging that SBE is used for skill development through spaced and experiential learning, a preferred skill acquisition and retention method [101,107]. While basic life support (BLS) and resuscitation simulations were common, studies on more complex simulations in clinical settings and non-medical professions are limited, revealing a gap in broader application. Though simulation for various skills is growing, its impact on clinical practice remains underexplored [88,109,155]. Assessing this impact is crucial to evaluating skills’ transferability and uncovering barriers that may hinder competence in real-world settings.

Community health is a significant area. Initiatives such as HBB and BLS training for lay people have been beneficial [85,108]. Integrating more simulation into community-based learning is crucial, especially in remote areas lacking healthcare access. Limited infrastructure for emergency care, such as ambulance services and trained emergency personnel, exacerbates mortality in roadside accidents and cardiac events, making expanded community training essential.

Activities Quality and Reporting: Is This Good Enough?

Some studies in our review were well-structured and rigorous, with strong data integration and analysis, enhancing their overall quality [24,72,164]. However, a few exhibited quality issues such as misclassifying study designs, reporting errors, providing insufficient details of simulation design, and not accounting for the initial exposure to simulation with subsequent educational interventions [54,58,68,84,121,130,137,138,160]. These flaws undermine their results’ rigor, replicability, and validity and hinder the development of evidence-based practices in simulation education [1,172].

Using a theory or framework is essential for scholarship [173]. While several studies applied appropriate frameworks, several relied on ad hoc approaches per their reporting [72,87,88,92,128]. The notion was supported as some authors identify the absence of dedicated, validated frameworks as a key barrier, while others highlight the benefits of using such models [24,69,84,154,164].

Our review identified several factors affecting the effectiveness of simulation in Pakistan. Key barriers include a lack of IRR due to rater fatigue, non-standardized checklists, and inadequate training [150]. Additionally, studies comparing simulation with traditional teaching observed a disconnect between learners’ perceived confidence and actual competence [98,129]. Addressing these issues will require better training for simulationists and the development of standardized assessment tools to enhance SBE.

Recommendations

To fully realize the transformative potential of SBE in Pakistan and similar LMICs, stakeholders across different levels must take coordinated action. Government policymakers, such as ministries of health and education, should allocate dedicated funding for the development of SBE infrastructure, focusing on equitable access for underserved rural and remote areas. They should also establish national policies to standardize simulation education and practices and integrate them into healthcare education frameworks. Institutional leaders, including heads of universities and training institutions, must champion faculty development programs to ensure educators are equipped with simulation-specific skills. They should integrate SBE into existing curricula and prioritize research on its long-term outcomes. International funding bodies and collaborators should support scalable, culturally adaptable simulation programs while investing in infrastructure and research capacity building in LMICs. Finally, researchers and educators must follow evidence-based practices and align their efforts to evaluate and improve the efficacy of SBE interventions, ensuring findings are accessible to stakeholders for evidence-based decision-making. Collaboration among all these groups is essential to overcoming financial, infrastructural, and training-related barriers, strengthening healthcare education, and improving patient outcomes.

Strengths

Our review had several strengths. We employed multiple search strategies and expanded the scope by including the local Pakistani database, PakMediNet [20]. The diversity of our research team further strengthened the study, including a healthcare librarian for thorough study inclusion and team members from varied cultural, educational, and professional backgrounds enriching the analysis. To maintain rigor, we upheld reflexivity through detailed note-taking, weekly meetings, and scholarly discussions, minimizing bias and ensuring objectivity. To maintain quality, two reviewers extracted data independently, with final decisions made by trained simulationists increasing the rigor (see IRR and robustness).

Limitations

Our review had certain limitations. The inconsistent terminology for SBE in Pakistan required us to use a broad range of search terms. Despite broadening the search criteria, some studies might have been missed. We did not conduct a risk of bias assessment, in line with scoping review guidelines [19], but mitigated subjectivity through a detailed journal and weekly discussions. Additionally, the review may have excluded relevant literature due to underdeveloped digital libraries, limited access to physical archives, and reliance on indexed databases. These additional factors could limit the comprehensiveness of the review, particularly for literature predating 2005, when simulation-related studies first appeared in digital databases. While we incorporated PakMediNet to include locally published studies, unpublished or gray literature, such as conference abstracts and dissertations, may have been overlooked. 

## Conclusions

This scoping review demonstrates that, while still developing, SBE in Pakistan has considerable potential to transform healthcare education. Key challenges include inconsistent terminology, insufficient faculty training, and limited focus on long-term skill retention. Despite these barriers, notable successes such as cross-institutional collaborations, internationally funded programs such as HBB, and innovative, low-cost solutions underscore the country’s adaptability. These findings are relevant not only to Pakistan but also to other LMICs facing similar socioeconomic conditions, healthcare needs, and training gaps.

While the recommendations address actionable solutions across various domains, this review highlights the need for cohesive efforts by policymakers, educators, and researchers to fully realize SBE’s transformative potential. Strategic investments in infrastructure, standardized practices for using simulation through better simulation education, and cost-effective tools must go hand-in-hand with rigorous evaluations and international collaborations. These steps will not only enhance healthcare education in Pakistan but also offer a replicable framework for advancing SBE in other LMICs, ultimately contributing to global healthcare equity and potentially improved patient outcomes.

## Appendices

Appendix A: original search strategy

Search Strategy 

((((((((((((((((((((((((((Computer simulation) OR (computer simulation[MeSH Terms])) OR (computer simulations[MeSH Terms])) OR (Simulation training)) OR (simulation training[MeSH Terms])) OR (Patient simulation)) OR (patient simulation[MeSH Terms])) OR (patient simulations[MeSH Terms])) OR (simulation)) OR (simulation[MeSH Terms])) OR (Medical simulation)) OR (medical simulation[MeSH Terms])) OR (simulation based medical education)) OR (simulation based medical education[MeSH Terms])) OR (High fidelity simulation training)) OR (high fidelity simulation training[MeSH Terms])) OR (Virtual reality)) OR (virtual reality[MeSH Terms])) OR (simulator)) OR (simulator[MeSH Terms])) OR (standardized patients)) OR (standardized patients[MeSH Terms])) OR (low fidelity simulation training)) OR (low fidelity simulation training[MeSH Terms])) AND (((((((((((((((((((((((((((((((((((((((((((((((Medical education) OR (medical education[MeSH Terms])) OR (Continuing medical education)) OR (continuing medical education[MeSH Terms])) OR (education, continuing medical[MeSH Terms])) OR (graduate medical education)) OR (graduate medical education[MeSH Terms])) OR (education, graduate medical[MeSH Terms])) OR (Internship and residency)) OR (intership and residency[MeSH Terms])) OR (Undergraduate medical education)) OR (undergraduate medical education[MeSH Terms])) OR (medical education, undergraduate[MeSH Terms])) OR (education, undergraduate medical[MeSH Terms])) OR (education, medical, undergraduate[MeSH Terms])) OR (teaching rounds)) OR (teaching rounds[MeSH Terms])) OR (residents)) OR (residents[MeSH Terms])) OR (Surgical specialties)) OR (surgical specialties[MeSH Terms])) OR (ophthalmological surgical procedures)) OR (ophthalmological surgical procedure[MeSH Terms])) OR (ophthalmological surgical procedures[MeSH Terms])) OR (procedure, ophthalmological surgical[MeSH Terms])) OR (procedures, ophthalmological surgical[MeSH Terms])) OR (cardiac surgical procedures)) OR (cardiac surgical procedure[MeSH Terms])) OR (cardiac surgical procedures[MeSH Terms])) OR (procedure, cardiac surgical[MeSH Terms])) OR (procedure, cardiac surgical[MeSH Terms])) OR (procedures, cardiac surgical[MeSH Terms])) OR (emergency medicine)) OR (emergency medicine[MeSH Terms])) OR (Delivery room management)) OR (delivery room[MeSH Terms])) OR (delivery room management[MeSH Terms])) OR (newborn evaluation)) OR (newborn evaluation[MeSH Terms])) OR (anesthesiology education)) OR (anesthesiology[MeSH Terms])) OR (radiology)) OR (hospital radiology department[MeSH Terms])) OR (pediatric surgery)) OR (pediatric surgery[MeSH Terms])) OR (community health care)) OR (care, community health[MeSH Terms]))) AND (Pakistan)) OR (pakistan[MeSH Terms])

Appendix B: modified search strategy 

PubMed (342)

(simulat*[tiab] or "standardized patients"[tiab] or "standardized participants"[tiab] or "augmented reality"[tiab] or "virtual reality"[tiab] or manikin*[tiab] or “cardiopulmonary resuscitation”[tiab] or “manikins”[mesh] or “simulation training”[mesh] or “computer simulation”[mesh]) and (educat*[tiab] or train*[tiab] or "professional development"[tiab] or "staff development"[tiab] or inservice[tiab] or teaching[tiab] or “inservice training”[mesh] or “education”[mesh] or “teaching”[mesh]) and (Pakistan*[tiab] or “Pakistan”[mesh] or “Pakistan”[Affiliation])

CINAHL (218)

(simulat* or "standardized patients" or "standardized participants" or "augmented reality" or "virtual reality" or manikin* or “cardiopulmonary resuscitation” or “MH "Simulations+" or MH "Models, Anatomic+") and (educat* or train* or "professional development" or "staff development" or inservice or teaching or MH "Professional Development+" or MH "Education+") and (Pakistan* or MH "Pakistan" or AF Pakistan)

PsycInfo (52)

(simulat* or standardized patients or standardized participants or augmented reality or virtual reality or manikin* or cardiopulmonary resuscitation or exp simulation/) and (educat* or train* or professional development or staff development or inservice or exp education/ or exp professional development/ or exp teaching/) and (Pakistan* or Pakistan.in,lo.)

PakMediNet (53)

The search function does not allow complex searches. Hence, each combination of terms from the simulation group and education group in the above strategies was searched.

Appendix C: references, aims, and relevant findings of the studies

**Table 11:** Appendix C: References, Aims and Relevant Findings of the Studies Abbreviations used in the table: GAGES - Global Assessment of Gastrointestinal Endoscopic Skills DOPS - Direct Observation of Procedural Skills ACE - Assessment of Competency in Endoscopy OSCE - Objective Structured Clinical Examination OSPE - Objective Structured Practical Examination SPIKES - Setting, Perception, Invitation/Information, Knowledge, Empathy, Summarize/Strategize OB GYN - Obstetrics and Gynecology CCCQ - Clinical Cultural Competency Questionnaire LMICs - Low and middle-income countries CMH - Combined Military Hospital SMART - Specific, Measurable, Achievable, Relevant, Time-bound

Note: The numbers at the end of the citations correspond to the numbers of the references in the manuscript.		

Reference	Aims of the study	Relevant findings reported
Ahmed M, Ahmad M, Malak A. Finding effectiveness of teaching basic life support to paramedics. Pakistan Armed Forces Medical Journal. 2014 Jun 30;64(2):225-8 [21].	To determine the impact of single setting basic life support courses to paramedics by analyzing their responses	The outcomes of training sessions over time can be improved with customized repeated courses to maximum individuals while stressing on practical application
Ahmed SI, Javed G, Mubeen B, Bareeqa SB, Rasheed H, Rehman A, Phulpoto MM, Samar SS, Aziz K. Robotics in neurosurgery: A literature review. Journal of the Pakistan Medical Association. 2018;68(2):258 [22].	To review the current data of recent robotic interventions in neurosurgery	Outcome reported: Different types of robotic devices are being used in neurosurgical procedures Outcomes met: The robots possess various levels of autonomy and different types of features, allowing their extensive use in neurosurgery.
Ahmed TM, Hussain B, Siddiqui MR. Can simulators be applied to improve cataract surgery training: a systematic review. BMJ Open Ophthalmology. 2020 Sep 1;5(1):e000488 [23].	This review assesses studies currently available that have evaluated the use of simulators in cataract surgery training.	Outcomes were classified as skill assessment, complication rate, skill acquisition and operating time.
Ahmed TM, Siddiqui AR. The role of simulators in improving vitreoretinal surgery training-A systematic review. Journal of Pakistan Medical Association. 2021 Jan 31;71(1):S-106 [24].	To conduct an appraisal of current evidence regarding the effectiveness of EyeSi®-based training of vitreoretinal surgery.	One study reported that EyeSi has concurrent validity and predictive validity by comparing the Global Rating Assessment of Skills in Intraocular Surgery (GRASIS) scores. Five studies reported that EyeSi has constructed validity by testing navigation training. One study reported that EyeSi has discriminated validity by looking at how distracting factors that are well known to result in a poor surgical performance led to decrease in performance scores on the simulator.
Ahsin S, Imran M, Fahim A, Hussain L. Student led outreach workshops to promote basic life support. Journal of the Pakistan Medical Association. 2021 Jul 1;71(7):1761-7 [25].	To provide basic life support training to the staff, students and faculty of higher educational institutions in urban areas through pre-trained medical students, and to record the qualitative impact of community training on student facilitators	Participants' pre-training knowledge improved across all areas, including checking the carotid pulse, providing rescue breathing, chest compressions, and ventilation. Medical students also reported greater self-confidence, enhanced basic life support (BLS) techniques, a stronger sense of responsibility toward the community, and a feeling of self-fulfillment.
Akber BA, Rajani MI, Khalid F, Docherty C. Simulated learning in rural community environment: pushing the boundary. Advances in Simulation. 2021 Dec;6:1-6 [26].	To discuss the concept of creating a novel simulated village set-up within a modern simulation center, to effectively deliver contemporary learning outcomes. To highlight the challenges and risks of developing a simulated village set-up and strategies to counteract them. To describe the role of simulation specialists as innovators and explicates the gamut of expertise in education, management, and technologies that are required to deliver excellence in simulation-based education.	Modern medical simulation centers use creative administrative and technical methods to create a realistic community setting, giving students practice and confidence before facing the actual environment. Organization of such an activity has allowed the team to develop their ability and start new novel simulation concepts that are rarely used.
Ali DM, Hisam B, Shaukat N, Baig N, Ong ME, Epstein JL, Goralnick E, Kivela PD, McNally B, Razzak J. Cardiopulmonary resuscitation (CPR) training strategies in the times of COVID-19: a systematic literature review comparing different training methodologies. Scandinavian journal of trauma, resuscitation and emergency medicine. 2021 Dec;29(1):1-6 [27].	We compared the learning outcomes of standard in-person CPR training (ST) with alternative methods of training such as hybrid or online-only training (AT) on CPR performance, quality, and knowledge among laypersons with no previous CPR training	Interactive-computer training combined with instructor-led practice resulted in higher mean scores for Cardiopulmonary resuscitation (CPR) performance compared to only computer-based training or only instructor-led training. The instructor-led training group performed better in quality of CPR compressions. No difference was found in CPR-related knowledge, with the video self-instruction group having better knowledge of average inflation volume and chest compression depth.
Ali KQ, Soofi SB, Hussain AS, Ansari U, Morris S, Tessaro MO, Ariff S, Merali H. Simulator-based ultrasound training for identification of endotracheal tube placement in a neonatal intensive care unit using point of care ultrasound. BMC Medical Education. 2020 Dec;20(1):1-1 [28].	To investigate the usefulness of a low-cost, novel point of care ultrasound (POCUS) simulator to train health care providers with minimal or no POCUS experience to accurately detect tracheal versus esophageal intubation in neonates. To investigate whether the time to POCUS image interpretation decreased with repeated simulator attempts.	The session effectively taught intubation point of care ultrasound (POCUS) to neonatal providers indicated by improved accuracy and time-to-interpretation with repeated use.
Ali L, Nisar S, Ghassan A. Role of debriefing as a learning tool in simulation based learning for students of preclinical years at the end of two consecutive modules-initial experience. Journal of Ayub Medical College Abbottabad. 2015 Jun 20;27(2):425-9 [29].	To analyze the usefulness of debriefing as an instructional strategy during observed structured clinical examination conducted at the end of two consecutive modules of first year MBBS students	Most students passed the second module objective structured clinical examination (OSCE) after the first debrief, suggesting its effectiveness. Students praised both debriefing sessions and suggested in-camera debriefing.
Ali MF, Nadeem N, Khalid F, Anwar NM, Nabie G, Docherty C. SonoGames: sounds of the right kind introducing gamification into radiology training. BMC Research Notes. 2021 Dec;14:1-7 [30].	To assess radiology residents’ knowledge, hands-on skills, and integration of knowledge into clinical decision making. It aims to evaluate simulation education (SE) of participants as a measure for competency using game-based (GB) simulation training programs.	Use of gamification in combination with simulation based teaching (SBT) improved post-test knowledge scores, positively affecting clinical training.
Ali S, Potokar TS, Chamania S, Lohana P, Price P, Whitaker IS. A novel, cost effective escharotomy simulator and trainee assessment. Burns-the Journal of the International Society for Burn Injuries. 2008 Jun 1;34(4):531-2 [31].	To improve morbidity and mortality of burns using escharotomy simulator	Simple escharotomy simulator was found to be a useful teaching adjunct. an increase in knowledge, and an increase in confidence to carry out the escharotomy procedure.
Amerjee A, Akhtar M, Ahmed I, Irfan S. Hybrid simulation training: An effective teaching and learning modality for intrauterine contraceptive device insertion. Education for Health: Change in Learning & Practice. 2018 May 1;31(2) [32].	To compare knowledge, procedural, and communication skills of medical students regarding Intrauterine Contraceptive Device (IUCD) insertion before and after introducing hybrid simulation training and to assess learner satisfaction with the new teaching methodology.	There was a significant increase in pre and post-simulation mean scores for all three skills; history-taking, counseling and procedural skills.
Arain MA, Begum S, Shariff AH, Khan S, Pal KI, Khan MR, Ali M, Ringers J. Prospective comparison of single encounter versus distributed laparoscopic training in novice learners: A controlled trial. Journal of Education and Health Promotion. 2022 Jan 1;11(1):116 [33].	To determine if acquisition and retention of laparoscopic skills were superior with multiple training encounters as compared to a single training session of similar duration and to identify the factors influencing learning laparoscopic skills in naïve medical students	Acquired skills decline over time. Repeated exposure and practice are needed to maintain dexterity.
Asghar MS, Zaman BS, Zahid A. Past, present, and future of surgical simulation and perspective of a developing country: A narrative review. Journal of the Pakistan Medical Association. 2021 Dec 1;71(12):2770-6 [34].	To highlight the historical aspects of simulations, their role in surgical education, and their importance in the future as an essential adjunct to surgical education.	-Initially, simulators made from routine materials like clay were used followed by benchtop models of wood, human cadavers and animal specimens. First-ever commercially produced manikins using different softwares along with microprocessors were produced afterwards. -Types of simulators included live animals, cadaveric simulators, bench-top and laparoscopically box-oriented simulators, virtual reality (VR) simulators, Robot-assisted surgery (RAS). -The most commonly applied surgical simulation in Pakistan includes laparoscopy simulators, box simulators, and VR simulators using a physics-based simulation, called Simulation Open Framework Architecture (SOFA).
Asif M, Shahzad N, Ali M, Zafar H. Teaching and practising rectal examination in Pakistan. The Clinical Teacher. 2015 Dec;12(6):399-402 [35].	To assess the attitude of final-year medical students and interns toward digital rectal examination (DRE) and to provide an overview of teaching practices.	Majority of of the participants appreciated the importance of the Digital Rectal Exam (DRE) and considered it as an essential skill. However, only half reported being taught about DRE before their final year of medical school.
Ather MH, Ng CF, Pourmand G, Osther PJ. Training the resident in percutaneous nephrolithotomy. Arab Journal of Urology. 2014 Mar 1;12(1):49-53 [36].	To examine the problems arising when training residents in percutaneous nephrolithotomy (PCNL) , and how to facilitate this process	-Steps considered difficult by residents include gaining access to the kidney, stone fragmentation, intracalyceal navigation, stone scavenging and fragmentation -Following steps should be taken: infrastructure optimization, expertise optimization, and financial optimization.
Ayaz O, Ismail FW. Healthcare simulation: a key to the future of medical education–a review. Advances in Medical Education and Practice. 2022 Apr 5:301-8 [37].	To provide relevant and comprehensive information regarding the utility of simulation in medical education and use knowledge theories to determine how to best implement it in under and postgraduate medical education	-Spectrum of healthcare simulation (HS) being used in medical education includes role playing, standardized patients (SP), part-task trainers, the computer patient, and the electronic patient/virtual reality (VR) -Rationale behind HS use in medical education includes the benefits of experiential learning for various scenarios especially during COVID-19. -Kolb’s learning cycle (four parts: concrete experience, reflective observation, abstract conceptualization, and active experimentation) has been used as a conceptual framework for HS in medical education. Apart from this, additional organizational steps including human capital assembly, integration into curriculum, deliberate practice encouragement are also required to implement HS.
Aziz A, Farhan M, Noor S, Alam S, Oduoye MO, Kamran AB. Virtual reality and simulation-based training in Pakistan for uniformity in neurosurgery training programs. Neurosurgical Review. 2024 Dec;47(1):1-3 [38].	To describe the current standard of neurosurgical training programs in Pakistan and the role of virtual reality (VR) and simulation-based programs in improving neurosurgical training	The training centres must integrate low-cost simulation models as well as VR/AR systems along with the conventional on-table didactic teaching, to cover all the basic aspects and provide equal high-quality training.
Aziz MM, Rasool MF, Alanazi M, Alharby TN, Alanazi J, Huwaimel B. Evaluating the counseling standards and ability of pharmacy staff to detect antibiotic-drugs interactions: A simulated client study from Pakistan. Antibiotics. 2023 May 19;12(5):931 [39].	The purpose of this research was to evaluate both the quality of antibiotic counseling provided and the level of understanding exhibited by pharmacy employees with regard to interactions involving antibiotic medications	-Community pharmacies lack in counselling skills as well as knowledge of antibiotic-drugs interactions (ADIs) as: 1)The rate of counseling without request was 3.6% which increased to 48% on demand 2) More than half of Simulated Clients (SCs) in both scenarios were advised to visit a physician, 3)Very few drug sellers advised the SC for medication change due to possible ADIs, 4)1.4% received special warnings or precautions about antibiotic use, 5)There was a scarcity of conversations regarding the potential adverse outcomes of therapy, 6)Lack of privacy, adequate duration of conversation, no focus on medication compliance, no information about drug storage were noted by the SCs.
Aziz MM, Yang S, Masood I, Zhu S, Raza MA, Ji W, Anwar N, Saeed A, Fang Y. An assessment of counseling quality provided by community pharmacies to type 2 diabetic adult patients for oral therapy: a simulated patient study from Pakistan. Endocrine Journal. 2019;66(3):233-9 [40].	To assess the quality of medicine counselling services provided by pharmacies in Pakistan to type 2 diabetic patients	Counseling provided to type 2 diabetic patients (DPs) in Pakistan's community pharmacies is subpar, with very few counsellings being provided without patient request. Furthermore, lack of privacy, lack of detail of disease, therapy reasons and side effects was also prevalent.
Azizi K, Ismail M, Aftab U, Afzal B, Mian A. Effectiveness of high-fidelity simulation in training emergency medicine physicians in point of care ultrasonography in Pakistan: A quasi-experimental study. Cureus. 2020 Jun 17; doi:10.7759/cureus.8659 [41].	To assess the knowledge and skills of emergency medicine (EM) physicians after a brief training workshop on e-FAST (Focused Assessment with Sonography in Trauma), including high-fidelity simulators, in a tertiary care teaching hospital in Karachi, Pakistan	Following training, all participants reported improvement in comfort and confidence with PoCUS, with the majority suggesting monthly ultrasound teaching sessions. Post-training scores increased significantly, indicating a high-fidelity simulation-based workshop improved emergency physicians' knowledge and skills.
Baig LA, Beran TN, Vallevand A, Baig ZA, Monroy-Cuadros M. Accuracy of portrayal by standardized patients: results from four OSCE stations conducted for high stakes examinations. BMC Medical Education. 2014 Dec;14:1-8 [42].	To assess the accuracy and realism of standardized patients (SP) portrayal as rated by experienced clinicians	-For cases A and D, SP portrayal demonstrated good internal consistency reliability but there was low reliability for Cases B and C due to inadequate view of camera angles. -Verbal portrayal by SPs did not significantly differ -Facial expressions of the SPs differed significantly -No major difference in portrayal at different time points during the OSCE -Highest range of variation was in cases requiring facial expressions Main conclusion drawn: overall rating of portrayal was “ok”
Bajwa M, Khatri A, Ali S, Ahmed R, Elgasim ME, Raechal L, Mukhtar S, Ansari T, Fayyaz J. Simplifying complexity science principles: developing healthcare faculty for using simulation as an educational method. International Journal of Healthcare Simulation. 2023; 1-12 [43].	To construct and deliver a professional development program using complex adaptive systems (CAS) theory to a Pakistani medical college faculty -To use simulation according to the Healthcare Standards of Best Practices.	Quantitative data: Participants reported improvements in acceptance, knowledge gain, self-efficacy, application of simulation knowledge, and facilitators' knowledge as a result of the activity Qualitative Data: Participants had a positive experience. Opinions on simulation concepts were mixed. Obstacles included lack of resources, time, and faculty. Leadership and management facilitated simulation-based education (SBE) implementation. Longer workshops were recommended.
Bakhshi SK, Ahmad R, Merchant AA, Noorali AA, Abdul Rahim K, Shaikh NQ, Afzal N, Lakhdir MP, Shamim MS, Haider AH. Development, outcome and costs of a simulation-based neurosurgery bootcamp at the national level. BMC Medical Education. 2022 Dec 28;22(1):896 [44].	To assess the effectiveness of structured boot camp on the knowledge and procedural skills of the participating neurosurgery residents (PGY1-Neurosurgery residents).	An overwhelmingly positive feedback was received, with nearly all activities rated greater than 4 for both relevance and usefulness on the Likert scale The trainers observed significant improvement in residents’ performance under supervision for all aspects of the cranial and spinal skills being assessed (assessment was only done for burr-hole, craniotomy, lumbar drain, laminectomy)
Bhutta SZ, Yasmin H. Comparative effectiveness of teaching obstetrics and gynaecological procedural skills on patients versus models: a randomized trial. Pakistan Journal of Medical Sciences. 2018 Jul;34(4):794 [45].	To determine whether utilization of mannequins and models for training interns in (Obstetrics and Gynaecology) OB/GYN in a structured program would result in knowledge and competencies comparable to those training on human subjects primarily, and up to the required standards for certification.	Initially, after 4 weeks no difference in the performance of the trainees was observed. However, after 8 weeks, trainees with experience on models and patients performed better, indicating better skill acquisition than those who practised only on patients.
Chagani SS, Aziz W, Farhan Z. Simulation‐based training module on Urosepsis improves knowledge and skills of urology nurses in Karachi, Pakistan: A brief project report. International Journal of Urological Nursing. 2022 Feb 23;16(3):252–7. doi:10.1111/ijun.12314 [46].	To evaluate the effect of knowledge and skills related to urosepsis of nurses after introducing a new simulation-based training initiative.	-The simulation-based training module significantly improved the knowledge and skills of urology nurses -The main strength of project was that participants learned hands-on skills to identify early warning signs of sepsis and to manage them efficiently and timely
Chaudhry AH, Bukhari F, Iqbal W, Nawaz Z, Malik MK. Laparoscopic training exercises using HTC VIVE. Intelligent Automation & Soft Computing. 2020 Mar 1;26(1) [47].	To simulate Laparoscopic skill exercises using HTC VIVE to give doctors a more real and 360 surgical environment and to assess factors and metrics that could enhance training or performance of participants.	-An improvement in performance of the students was observed after the final trial
Dias J, Zuberi RW. Establishing a simulation centre in Karachi, Pakistan. COJ Nursing & Healthcare. 2018;1(4) [48].	To describe a state-of–the–art, multipurpose, multidisciplinary and multi-professional facility for doctors, nurses, dentists and allied health personnel to enhance cognitive, clinical, behavioral, communication and procedural skills.	Nearly all important steps should be initiated before the inauguration, for example, a Steering Committee to lay down guidelines, a Director for implementation etc. -To develop such an institute, individuals from all fields including medicine, nursing, Information-Technology, dentistry, biomedical engineering, Marketing, Design-office, Finance, Security, Food Services, Facilities and Material Management should be taken board
Docherty C, Rajani M, Shahpur N, Khalid F, Ukrani RD. Mobile healthcare simulation units: a narrative review. Journal of Pakistan Medical Association. 2023 Dec 31;73(12):2403- [49].	To assess the emergence and use of Mobile Healthcare Simulation Unit (MHSU) globally while investigating the value of simulation-based education (SBE) to those remote and rural HCPs who have benefitted from having access to their services, and gauging their satisfaction. To identify pros and cons of developing an MHSU, to indicate which of the approaches are more efficient and effective than the others, to identify key performance indicators (KPIs) and outcome measures to indicate value and worth of an MHSU.	MHSU included adapted trucks, hybrid MHSUs which can be used for SBT and treatment of real patients, inflatable mobile simulation environments Majority of the participants and teaching faculty reported high satisfaction of MHSU. It reduces cost of simulation and provides interprofessional training for both skills and knowledge. Lack of continuous funding can be dealt through with simulator sharing, lack of adequate staffing can be overcome through liaison hospital, lack of a multipurpose design which can be overcome by building MHSUs on new chassis with a more flexible approach and to store MHSUs in buildings to prevent weather damage.
Farooq S, Tharani A, Begum S, Parpio Y. Implementation of simulation: A contemporary strategy to enhance clinical skills of undergraduate students in mental health nursing. Issues in Mental Health Nursing. 2020 Aug 2;41(8):736-40 [50].	To determine the significance of simulation to improve the communication skills and confidence level, and to decrease the anxiety of undergraduate nursing students, prior to attending mental health clinical.	The students’ formative feedback and summative evaluation on the simulation-based teaching learning strategy was positive
Farrukh K, Zehra F, Shahid F, Mehr Y. Health sciences faculty attitude and readiness towards simulation-based inter-professional education. Medical Education. 2024 Feb [51].	To explore attitude and readiness of health sciences faculty towards simulation-based inter-professional education (IPE).	Faculty members value inter-professional education (IPE) but are reluctant to implement it. There is a need for faculty development for implementation of simulation-based IPE. There is insufficient research on simulation and IPE in Pakistan because of which faculty members are unacquainted of its benefits.
Fayyaz J, Jaeger M, Takundwa P, Iqbal AU, Khatri A, Ali S, Mukhtar S, Saleem SG, Whitfill T, Ali I, Duff JP. Exploring cultural sensitivity during distance simulations in pediatric emergency medicine. AEM Education and Training. 2023 Dec;7(6):e10908 [52].	To explore the impact of a 6-week-long synchronous distance simulation curriculum in promoting cultural sensitivity (CS) among Pakistani Emergency Medicine (EM) learners and U.S. pediatric emergency medicine (PEM) fellow facilitators from diverse cultural backgrounds	-Pre-training CCCQ assessment revealed that all learners thought that it was imperative to have training about cultural sensitivity (CS) in undergraduate and postgraduate curricula. While most of the Pakistani EM residents had no access to such training during their residency training, most U.S. pediatric emergency medicine (PEM) fellows had some training during their residency. -Qualitative analysis revealed three themes: 1)Learning experience (both groups found the training helpful) 2)Intercultural encounter (subthemes include ethnic/cultural diversity, linguistic diversity, cultural aspects surfaced in training, power distance) 3)Challenges (subthemes include technical aspects, scenarios, facilitation, debriefing)
Ghauri SK, Khan H, Bangash MA, Mustafa KJ, Khan AS. Impact of basic life support training on the knowledge of basic life support in undergraduate medical students. South Asian Journal of Emergency Medicine. 2020 Apr 19;1(1):03- [53].	To assess the impact of basic life support (BLS) training on the knowledge of a group of undergraduate medical students.	This study has shown a deficiency in the knowledge of BLS among medical students with a significant increase following a BLS training workshop.
Ghazanfar S, Qureshi S, Zubair M, Safdar Y, Leghari AA, Quraishy MS. Is laparoscopic experience helpful in simulator based robotic training in general surgery?Journal of Pakistan Medical Association. 2021;71(2198) [54].	To evaluate whether or not prior laparoscopic training improves performance during robotic surgery utilising DaVinci robotic skills simulator.	Laparoscopic skills did not confer any benefit while performing exercises on the DaVinci skills simulator as no significant difference in the scores of the two groups was observed.
Gul M, Arif A, Ghafoor R. Role of three-dimensional printing in periodontal regeneration and repair: Literature review. Journal of Indian Society of Periodontology. 2019 Nov 1;23(6):504-10 [55].	To document all the English language literature regarding applications of 3D printing in periodontology after detailed and thorough literature search.	Literature reports the following use of 3D printing: 1)3D printed bioresorbable scaffold for guided bone and tissue regeneration 2)Socket preservation 3)Sinus and bone augmentation 4)Three-dimensional printing for implants placement 5)Use of three-dimensional printing for peri-implant maintenance 6)Use of 3D printing for implant education
Hafeez M, Sohail N, Waqar SH. Perceptions of trainees about use of simulation in gynaecology and obstetrics. Pakistan Armed Forces Medical Journal. 2021 Dec 30;71(6):2001-06 [56].	To explore the perception of Obstetrics and Gynaecology (OB/GYN) postgraduates about the use of simulation in improving their clinical skills.	-There was a significant improvement in clinical performance after training -Simulation allows practising and gaining an optimal level of skill in a safe and protected learner-centred environment. -4 themes were identified from the qualitative data: 1) Clinical experience before simulation (lack of confidence, fearfulness, procedural mistakes, lack of experience), 2) Clinical experience after simulation (acquired confidence, obtained comfort ability, acquired skillfulness and experience), 3) Perceived benefits of simulation (time friendliness, source of deliberate practice, safe practice), 4) Suggestions (improvement of ease of access of simulation, adjunct material to improve learning)
Haji Z, Arif A, Jamal S, Ghafoor R. Augmented reality in clinical dental training and education. Journal of the Pakistan Medical Association. 2021;71(1 (Suppl 1)):S42 [57].	-	The benefits of virtual reality (VR) with the support of augmented reality (AR) are clearly evident.
Hajira B, Rana A, Naeem F, Qammer Y. Virtual skills teaching of nursing students during COVID-19: A problem-solving Approach. Journal of College of Physicians and Surgeons Pakistan. 2022 Apr 1;32(4):548-9 [58].	To give an overview and practical application of alternative solutions to overcome the obstacle of COVID-19 impeding clinical nursing education	This initiative enhanced students' self-directed learning, confidence to perform the above skills efficiently, and satisfaction while performing skills on their family members.
Hamid SS, Zehra T, Tariq M, Afzal AS, Majid H, Hussain E. Implementing work place based assessment: The modified direct observation of procedural skills (DOPS) across medical specialties-An experience from a developing country. Journal of the Pakistan Medical Association. 2022 Apr 1;72(4):620-4 [59].	To assess the skill level of residents regarding central venous catheterisation insertion, and to assess the reliability of scores in a simulated situation.	There was significant improvement in the skills level post-intervention compared to the baselines mean values. The subjects were satisfied with the workshops
Haque A, Arif F, Abass Q, Ahmed K. Development of pediatric neurologic emergency life support course: A preliminary report. Pediatric Emergency Care. 2017 Nov 1;33(11):e114-7 [60].	To educate first level of healthcare providers on the first-hour management of children with acute neurological emergencies (ANEs)	This course enhanced the knowledge and boosted the confidence of the participants.
Haroon Z, Azad AA, Sharif M, Aslam A, Arshad K, Rafiq S. COVID-19 era: challenges and solutions in dental education. Journal of College of Physicians and Surgeons Pakistan. 2020 Oct 1;30(10):129-31 [61].	To discuss the impact of COVID-19 on dental education along with some solutions to these challenges	-COVID-19 greatly impacted dental education by halting continued dental education with a reduced number of prospective dental students and increasing rate of college dropouts. -Innovations in dental education to deal with these challenges include electronic platforms for e-learning, online assessment strategies, pre-clinical and clinical learning, dental research activities, curriculum amendments and psychological support to both students and faculty
Hasan O, Iqbal S. A framework for laparoscopic simulations. Journal of Pakistan Medical Association. 2017;67(9):1404-1409 [62].	To provide a concise framework of a laparoscopic simulator that is based on solid scientific principles and captures most of the training requirements of a laparoscopic surgeon.	The developed simulator (SmartSIM) was based on the above framework with certain modifications including the features of trainee assessment and improving motor skills.
Hasan OH, Atif M, Jessar MM, Hashmi P. Application of 3D printing in orthopaedic surgery. A new affordable horizon for cost-conscious care. Journal of the Pakistan Medical Association. 2019;69(Supl. 1):S46 [63].	The current literature review was planned to highlight the advantages of using 3D printing, its scope in surgical field with emphasis on orthopaedic surgery, and the limitations of its use in developing countries.	Application of 3D printing in orthopedic surgery include: 1)Conversion of medical images into 3D objects 2)Surgical planning 3)Medical education and training 4)Implants 5)Casts
Hasan OH, Ayaz A, Khan M, Docherty C, Hashmi P. The need for simulation in surgical education in developing countries. The wind of change. Review article. Journal of the Pakistan Medical Association. 2019;69(Supl. 1):S62 [64].	To go over the importance of simulation in surgical training and to emphasize the need to integrate this technology into surgical practice in developing countries.	-Simulation is necessary for surgical training as it allows repetitive practice of various procedures thereby greatly reducing the learning curve. Additionally, lack of access to surgical care, lack of training facilities, and increased perioperative complications further highlight the need for surgical simulation.
Hussain A, Ibrahim MI, Malik M. Assessment of disease management of insomnia at community pharmacies through simulated visits in Pakistan. Pharmacy Practice. 2013 Oct;11(4):179 [65].	To document and compare the process of case management for insomnia at community pharmacies in three major areas of Pakistan namely Islamabad (national capital), Peshawar (capital of Khyber Pakhtunkhwa province) and Lahore (capital of Punjab province)	Overall, the process of case management of insomnia at community pharmacies in Pakistan is not satisfactory. There was no significant difference in the case management of insomnia at community pharmacies located in different settings in the three cities.
Hussain A, Mirza Z, Qureshi FA, Hafeez A. Adherence of private practitioners with the National Tuberculosis Treatment Guidelines in Pakistan: a survey report. Journal of Pakistan Medical Association. 2005 Jan 1;55(1):17-9 [66].	To evaluate the adherence of private practitioners with tuberculosis (TB) treatment guidelines as laid down by National Tuberculosis Control Programme (NTP) in Pakistan	Inappropriate prescribing is prevalent among GPs, with very few prescriptions meeting the National Tuberculosis Program (NTP) guidelines. Additionally, pyridoxine, an essential supplement, was rarely prescribed and most of the prescriptions contained inappropriate and irrelevant medications like ciprofloxacin, doxycycline, and clarithromycin.
Hussain S, Rana RE, Ather MH. Validation of a bench-top training model for retrograde intrarenal surgery. Urologia Internationalis. 2021 Jun 30;105(7-8):605-10 [67].	To determine the face, content, and construct validity of the in-house built Styrofoam box bench-top training model for retrograde intrarenal surgery (RIRS)	The present study has demonstrated face, content, and construct validity of an easily reproducible and very cheap bench-top model for RIRS training.
Inam H, Asif N, Sohail AA, Fatimi SH. Wet labs: A useful tool in training surgical residents in a third world country. Annals of Medicine and Surgery. 2020 Sep 1;57:137-9 [68].	This letter to the editor details the technicalities of a wet lab, its advantages. What it takes and how it is done. The technical proof of the concept is the first phase in development of wet labs as an integral part in the curriculum of residents learning.	Wet labs are a cost-effective teaching tool that boost residents' confidence and consultants. It helps teach advanced surgical skills, particularly in resource-limited settings where expensive simulators are unavailable.
Inayat S, Younas A, Sundus A, Khan FH. Nursing students' preparedness and practice in critical care settings: A scoping review. Journal of Professional Nursing. 2021 Jan 1;37(1):122-34 [69].	To map and analyze the literature about nursing students' placement, preparedness, and practice in critical care settings and identified areas for future research and practice.	1)Theme I: students' experiences of critical care placements; -Subtheme 1: Importance of and need for critical care placements; -Subtheme 2: Challenges encountered during clinical placement and practice 2)Theme II: teaching and learning strategies to enhance student learning; -Subtheme 1: course based teaching; -Subtheme 2: mentorship & preceptorship; -Subtheme 3: problem and team based learning; -Subtheme 4: simulation 3)Theme III: impact of clinical placements and teaching strategies on students; -Subtheme 1: confidence; -Subtheme 2: competence; -Subtheme 3: communication
Iqbal M, Khizar B, Zaidi Z. Revising an objective structured clinical examination in a resource-limited Pakistani medical school. Education for Health. 2009 Apr 1;22(1):209 [70].	To describes the experiences and perceptions of our faculty and students with changes in OSCE format, within a resource-limited environment	Both the students and the faculty members greatly appreciated the OSCE format, and considered it important for developing various skills required in medicine
Iqbal S, Ahmed W. Innovation in surgical training: Simulating prosthetic heart valves and annuloplasty rings using pacifiers for cardiac surgery wet-labs in a resource limited setting. Journal of the Pakistan Medical Association. 2021 Jan 1;71(1):S124-6 [71].	The current short report presents a novel simulator to mimic heart valve replacement surgeries by using a pacifier as a prosthetic valve and annuloplasty ring to facilitate other resource-limited residency programmes	Regular wet-labs are an effective training strategy for cardiac surgery residents, but limited in LMICs due to financial constraints. Bovine hearts, basic instruments, and pacifiers can be used in resource-limited settings for successful wet-labs, perfecting valve surgery skills.The use of pacifiers is recommended as a cheap, durable, easily-available and easily-assembled simulator for perfecting the art of valve surgeries.
Ismail FW, Afzal A, Durrani R, Qureshi R, Awan S, Brown MR. Exploring endoscopic competence in gastroenterology training: A simulation-based comparative Analysis of GAGES, DOPS, and ACE assessment tools. Advances in Medical Education and Practice. 2024 Dec 31:75-84 [72].	To compare three different assessment tools to identify the tool that most accurately reflects competence in endoscopic skills	The study demonstrated that all three assessment tools were found to have their advantages and drawbacks, and it would be inappropriate to pick one as the best for all contexts.
Jabeen D. Use of simulated patients for assessment of communication skills in undergraduate medical education in obstetrics and gynaecology. Journal of College of Physicians and Surgeons Pakistan. 2013 Jan 1;23(1):16-9 [73].	To compare the effectiveness of simulated patients with real patients through undergraduate students' results of (Mini-Clinical Evaluation Exercise) Mini-Cex encounters and their opinions	Undergraduate students were more in favour of using simulated patient encounters for evaluation of communication skills. There was no significant difference between students' performance on real and simulated patients.
Jawaid M, Baig L, Aly SM. Comparison of OSCE scores of surgical clinical education after face-to-face vs. blended learning methods among undergraduate medical students. Journal of Pakistan Medical Association. 2021 Jun 1;71(6):1535-9 [74].	To compare the efficacy of blended learning with contemporary face-to-face teaching among medical students.	OSCE scores for the blended learning (BL) group were significantly higher than those for the face-to-face (F2F) group.
Kamal MM, Sohail AA, Qureshi NJ, Ullah H, Shahabuddin S, Kamal MY. Simulating aortic root replacement for a surgical wet laboratory in a resource-limited setting: an economical innovation. Interdisciplinary Cardiovascular and Thoracic Surgery. 2023 Oct 1;37(4):ivad167 [75].	To introduce an innovative and cost-effective way of simulating aortic root replacement in a wet laboratory by applying a hand- made valve conduit or ‘pencil conduit’ to a bovine heart.	The simulation model is cost effective, simple and quickly assembled. It allows trainees and faculty members to receive training in a resource-limited setting to practice an advanced procedure safely.
Kanwal L, Gulzar M, Idrees W, Ikram F, Sukhia RH, Fida M. The application of virtual reality and augmented reality in dentistry-a literature review. Journal of the Pakistan Medical Association. 2024 Apr 1;74(4 (Supple-4)):S126-31 [76].	To present the latest advancements, document the continuous evolution of augmented reality (AR) and virtual reality (VR) in diverse areas of dentistry, and pinpoint areas requiring additional research to facilitate their practical implementation in clinical settings.	-AR and VR are being employed in the following fields: restorative dentistry and endodontics, orthodontics, prosthodontics, oral and maxillofacial surgery, pediatric dentistry, dental implantology -Further research is required in use of VR/AR in diverse dental procedures and conditions of social distancing
Kashif L, Mahboob U, Ahmed F. Prioritization of factors by medical faculty towards introduction of centralized objective structured clinical and practical examination Khyber Medical University Journal. 2021 Jun 30;13(2):108-2 [77].	To prioritize the factors required for the implementation of the centralized, Objective Structured Clinical Examination (OSCE) and Objective Structured Practical Examination (OSPE) to standardize the clinical examination across the province of Khyber Pakhtunkhwa, Pakistan. This study was aimed to identify the perceptions of medical faculty of public and private medical colleges from Peshawar, Pakistan towards the introduction of centralized OSCE/OSPE, factors affecting its implementation and challenges towards its introduction.	-Majority of the participants not only did not have proper training in organizing, conducting and developing OSCE/OSPE stations but also lacked any proper training in assessments and marking schemes. - most of the participants did not emphasize regular training and recruitment of standardized patients and examiners. One of the reasons for this is the rapid changeover of the faculty job placements and ignorance considering the importance of standardization. -To develop uniformity in the OSCE/OSPE, development of a central OSCE/OSPE bank, central monitoring by the assessing university, and provision of standard stations, was highly prioritized by the participants -Majority of the participants gave low prioritization to appropriate financial support despite believing that having adequate human resources is imperative for a uniform OSCE/OSPE conduction.
Khaliq T, Atif QA. Simulation in surgery. Journal of College of Physicians and Surgeons Pakistan. 2019 Mar 1;29(3):199-200 [78].	The level of diffusion of simulation based training in the field of surgery	NR
Khan A, Amerjee A, Dias JM, Tariq J. From tradition to Simulation: An experience of team training on management of shoulder dystocia. Journal of the Pakistan Medical Association. 2022;72(1):47 [79].	To determine if simulation-based team training improves the management of shoulder dystocia compared to traditionally taught obstetrical emergencies	Training of serious obstetrical emergencies with simulation-based team training in the simulation lab was found to be superior to the traditional methods. It is an excellent platform for deliberate practice, and reflection on one’s own action to develop competence.
Khan HS, Sheikh NS. Role-play: A simulated teaching technique in physiology. Pakistan Journal of Physiology. 2021 Dec 31;17(4):46-50 [80].	To record and assess the response of MBBS undergraduate students to role-play of clinical scenarios during the teaching of Physiology at CMH Lahore Medical College and Institute of Dentistry.	A majority of students have expressed a positive response towards role play as a teaching methodology in the teaching of clinical scenarios in Physiology
Khan JA, Kiani MR. Impact of multi-professional simulation-based training on perceptions of safety and preparedness among health workers caring for coronavirus disease 2019 patients in Pakistan. Journal of Educational Evaluation for Health Professions. 2020;17 [81].	To determine whether health workers’ perceived levels of preparedness, safety, and willingness to care for COVID-19 patients changed after attending the simulation-based course.	Multi-professional simulation-based training imparted confidence and a sense of preparedness among health workers to care for patients with infections that require strict isolation, such as COVID-19.
Khan JA, Shafquat A, Kundi A. Basic life support skills: assessment and education of spouse and first degree relatives of patients with coronary disease. JJournal of College of Physicians and Surgeons Pakistan. 2010 May 1;20:299-302 [82].	to assess the knowledge of basic cardiac life support among spouses and first degree relatives of patient with CHD followed by training and reassessment of knowledge about basic life support	There was significant improvement in relatives’ knowledge of Basic Life Support (BLS), after training. Different age groups, both genders and all educational groups showed equal learning abilities
Khan MR, Begum S. Apprenticeship to simulation: the metamorphosis of surgical training. Journal of Pakistan Medical Association. 2021;71(Suppl 1)(1):S72-S76 [83].	To describe the evolution and transformation of surgical training over time.	The concept of "see one, do one, teach one" has widened its horizons to inculcate simulation-based training of both technical and non-technical skills that have direct impact on patient outcome.
Khan MR, Shariff AH, Nasim S, Sayyed RH, Effendi MS, Pinjani S. Effectiveness of laparoscopic skills workshop on enhancing knowledge and skills of surgical residents and its comparison with DOPS (Direct Observation of Procedural Skills) scores: prospective cohort study. Medical Science Educator. 2020 Jun;30:861-7 [84].	The aim of our study was to explore if a 1-day laparoscopic skills workshop enhanced the knowledge and skills of surgical residents in minimal access surgery and if it had any correlation with the Direct Observation of Procedural Skills (DOPS) scores.	One-day laparoscopic skills workshop resulted in significant improvement in knowledge and psychomotor skills of the surgical residents. The skills gained from the workshop also resulted in improvement of Direct Observation of Procedural Skills (DOPS) scores reflecting the transfer of skills to real-life performance
Khan UR, Khudadad U, Baig N, Ahmed F, Raheem A, Hisam B, Khan NU, Hock MO, Razzak JA. Out of hospital cardiac arrest: experience of a bystander CPR training program in Karachi, Pakistan. BMC Emergency Medicine. 2022 Jun 3;22(1):93 [85].	To determine the retention of knowledge and skills of Hands-Only CPR training among community participants in early recognition of Out of Hospital Cardiac Arrest (OHCA) and initiation of CPR in Karachi, Pakistan.	The bystander cardiopulmonary resuscitation (CPR) training program did not only improve the knowledge and skills of the participants but also had a reasonable retention rate.
Khan ZA, Kamal N, Hameed A, Mahmood A, Zainab R, Sadia B, Mansoor SB, Hasan O. SmartSIM‐a virtual reality simulator for laparoscopy training using a generic physics engine. The International Journal of Medical Robotics and Computer Assisted Surgery. 2017 Sep;13(3):e1771 [86].	To describe a new virtual reality (VR) simulator for basic training in laparoscopy, i.e. SmartSIM, which has been developed using a generic open‐source physics engine called the simulation open framework architecture (SOFA). This paper describes the systems perspective of SmartSIM including design details of both hardware and software components, while highlighting the critical design decisions.	The SmartSIM is superior to box trainers and commercial simulators in minimal invasive surgical (MIS) skills training due to its higher usefulness, realism, and improved graphics quality. It also offers reduced training costs, practice of unusual medical scenarios, quick assessment of laparoscopic surgical skills, and ease of mobility.
Kurji Z, Aijaz A, Aijaz A, Jetha Z, Cassum S. Telesimulation innovation on the teaching of SPIKES Model on sharing bad news. Asia-Pacific Journal of Oncology Nursing. 2021 Nov 1;8(6):623-7 [87].	To document how the faculty planned and implemented the Telesimulation (TS) strategy during COVID-19	Challenges faced by SPs are emotional burden from portraying terminal illness, information technology (IT) issues causing connection difficulties, and mental fatigue from repeated scenarios. Challenges faced by Trainee nurse interns (TNI): depression from personal family experiences, preference for conducting sensitive activities without peers, difficulty conveying empathy in virtual settings.
Liaqat M, Hussain M, Afzal M, Altaf M, Khan S, Gilani SA, Liaqat I. Efficacy of pedagogical framework in neonatal resuscitation skill learning in a resource-limited setting: a randomized controlled trial. BMC Medical Education. 2021 Dec;21:1-0 [88].	To compare the technical and non-technical skills among undergraduate nursing students learning neonatal resuscitation through “Learn, See, Practice, Prove, Do, Maintain (LSPPDM) pedagogy” compared to those who had learned through the traditional method	A significantly positive effect was observed in context with the technical and non-technical skills of nursing students after the intervention.
Mahdi SS, Jafri HA, Allana R, Battineni G, Khawaja M, Sakina S, Agha D, Rehman K, Amenta F. Systematic review on the current state of disaster preparation Simulation Exercises (SimEx). BMC Emergency Medicine. 2023 May 24;23(1):52 [89].	To investigate the current state of SimEx practices in disaster preparation, including their types, usage frequency, and effectiveness To determine how far SimEx can work as an important educational tool for disaster preparedness to provide the desired outcome of enhanced field performance as well as obstacles and patterns in SimEx use	-SimEx is currently being used for following exercise types: drill exercise, full-scale exercise, tabletop exercise, workshop discussion-based simulation exercises, computer-based simulation exercise, operational-based functional exercises. -Effectiveness of different type SimEx was evaluated by following methods: questionnaires/Likert scales, debriefing pre session, post-exercise exam, follow-up interviews. cognitive task analysis, post-exercise Homeland security and Exercise Evaluation Program (HSEEP) participant feedback forms, and Incident Command (IC) specific exercise evaluation guide
Mahmud T, Nasir T, Saqib M, Aasim M, Siddique N. Simulation training using cadaver sheep chest in pleuroscopy—A step towards skills enhancement. Journal of the Pakistan Medical Association. 2017 Apr 1;67(4):552-5 [90].	For assessing the use of simulation training on animal cadavers as a useful tool for training in pleuroscopy	All the respondents uniformly accepted the utility of simulation training in enhancing education, improving skill, and improving confidence by repeated practice, and felt that the inclusion of animal models for learning fundamental pleuroscopic procedures can help a lot in teaching
Malik AA, Ayyaz M, Afzal MF, Ali AA, Shamim R, Khan R, Khan HS, Naeem A, Bhatti S. Use of box simulators for improving intraoperative laparoscopic skills-an essential tool for the surgeon in training. Journal of the College of Physicians and Surgeons of Pakistan 2015 Mar 1;25:172-5 [91].	To compare the mean increase in global assessment of laparoscopic skills (GOALS) score measuring intraoperative laparoscopic skills between residents who have undergone simulator training for 16 hours with those who have not received any simulator training	Both groups showed improvement in their GOALS score however, the simulator trained group improved significantly more than the non-intervention group.
Malik MU, Connelly TM, Awan NA, Bhatti FR, Hayat RS, Awan HR. The development and evaluation of a homemade laparoscopic endotrainer using the IDEAL framework and MISTELS scoring system: a pilot study. ANZ Journal of Surgery. 2024 Feb;94(1-2):84-8 [92].	To evaluate the reliability of a homemade laparoscopic endotrainer, following the McGill inanimate system for training and evaluation of laparoscopic skills (MISTEL) and Idea, Development, Exploration, Assessment and Long-term follow-up (IDEAL) framework guidelines.	The MISTELS validated scoring system demonstrated reliability.
Malik OA, Chhapra R. An Inexpensive model to teach hemorrhage control in resource limited settings. Pakistan Journal of Medical Sciences. 2021 May;37(3):916 [93].	To describe a simple, low – cost alternative to commercially available mannequins to teach hemorrhage control using simulated scenarios.	Majority of participants felt confident in their improved ability to control life-threatening hemorrhage both in and out of the hospital
Malik S, Hasan S, Hamad A, Khan H, Bilal M. Conventional/ traditional practical examination (CPE/TDPE) versus objective structured practical evaluation (OSPE)/semi objective structured practical evaluation (SOSPE). Pakistan Journal of Physiology. 2009 Jun 30;5(1) [94].	To assess the validity of objective structured practical evaluation (OSPE) over Traditional Practical Examination (TDPE) as a system of assessment	-The mean score of Traditional Practical Examination (TDPE) was found to be significantly higher than that of objective structured practical evaluation (OSPE) but like others the correlation was weak indicating that they both test different abilities. The student's attitude towards OSPE was found to be positive
Malik S, Zaheer R, Bilal M. Impact of movie-based simulation training, with or without conventional verbal demonstration on observed OSPE scores in medical undergraduates: a double control study. Journal of Ayub Medical College Abbottabad. 2013 Jun 1;25(1-2):127-8 [95].	To compare the movie-based and traditional verbal demonstration teaching methodologies through objective structured practical evaluation (OSPE) scores.	Even half an hour of movie-based simulation training with traditional-instructor-based training can improve the student performance significantly.
Malik TG, Mahboob U, Khan RA, Alam R. Virtual patients versus standardized patients for improving clinical reasoning skills in ophthalmology residents. A randomized controlled trial. BMC Medical Education. 2024 Apr 22;24(1):429 [96].	Primary objective was to determine the effectiveness of using the virtual patient (VP) (as an educational intervention) on the clinical reasoning skills of ophthalmology residents, as measured by pretest and posttest scores. Secondary objective was to evaluate the retention of clinical reasoning by repeating the posttest after one month	Clinical reasoning improved greatly with virtual patients (VPs) and standardized patients (SPs). The VP and SP groups' end results were not statistically significant, demonstrating that both methods were equally effective at teaching ophthalmology residents clinical reasoning. Additionally, net gain in clinical reasoning skill from pretest to follow-up posttest.
Malik UR, Chang J, Hashmi F, Atif N, Basir H, Hayat K, Khan FU, Kabba JA, Lambojon K, Fang Y. A simulated client exploration of nonprescription dispensing of antibiotics at drugstores for pediatric acute diarrhea and upper respiratory infection in Lahore, Pakistan. Infection and Drug Resistance. 2021 Mar 22:1129-40 [97].	To assess the nonprescription antibiotics dispensed in children at drugstores of the capital city of Punjab province of Pakistan, using the simulated client method	The malpractice of dispensing non-prescription antibiotics is highly prevalent at the drugstores of Lahore, Pakistan. Most of the antibiotics were dispensed by non-qualified and less- trained staff and also not according to standard disease management guidelines.
Martins RS, Sabzwari S, Iqbal M. Effectiveness of simulation-based clinical skills training for medical students in respiratory medicine: a pilot study. Journal of the College of Physicians and Surgeons of Pakistan. 2021;31(12):1468 [98].	To assess the effectiveness of high-fidelity simulation-based medical education (HF-SBME) in teaching and learning respiratory clinical examination in medical students	Although medical students perceived high-fidelity simulation-based medical education (HF-SBME) as a beneficial teaching modality, it did not translate into improved performance.
Masood Z, Qabool H, Fida M, Sukhia RH. Exploring the knowledge and awareness on applications of virtual reality and augmented reality technology among dental healthcare professionals-a crosssectional survey. Journal of the Pakistan Medical Association. 2024 Apr 1;74(4 (Supple-4)):S10-6 [99].	The primary objective of this study is to assess the difference in awareness and utilization of virtual reality (VR) and augmented reality (AR) technology among dental healthcare professionals, including dentists, dental educators, and dental students. The secondary objective is to determine the acceptance of dental healthcare professionals to incorporate VR and AR into their educational and clinical practices.	-Dental healthcare professionals lack adequate levels of awareness of augmented reality (AR) and virtual reality (VR) technology for seamless integration into education and practice however, the majority expressed positive views about the potential impact of AR/VR technologies on the future of dental healthcare.
Mazhar MA, Qazi S, Sarwat S. The future of anatomy education: Simulation-based and AI-based learning. Journal of Clinical Nursing. 2024 Feb 14 [100].	To describe the future impact of simulation on anatomical education.	NR
Mehmood MH, Siddiqi S, Rehman R. Medical simulator, an innovative tool for experiential learning, application and reflection of knowledge. Journal of the Pakistan Medical Association. 2018;68(7):1142 [101].	To provide evidence to the usefulness of medical simulators as innovative and worth adding tools for deep and student-centered learning.	A simulation-based learning blueprint in medical education includes developing clinical scenarios, pre-briefing students with SMART objectives, displaying the scenario, conducting interactive case analysis to diagnose and rationalize treatment, and concluding with a debriefing session for reflection and application of the acquired knowledge.
Menna G, Kolias A, Esene IN, Barthélemy EJ, Hoz S, Laeke T, Silva AC, Longo-Calderón GM, Baticulon RE, Zabala JP, Hassani FD. Reducing the gap in neurosurgical education in LMICs: a report of a non-profit educational program. World Neurosurgery. 2024 Feb 1;182:e792-7 [102].	The present paper aims (1) to evaluate the validity and usability of a cadaver-free hybrid system in the context of Low- and middle income countries (LMICs) and (2) to report their learning needs and whether the courses meet those needs via a comprehensive survey	A cadaver-free hybrid training system could help in accelerating the learning curve of neurosurgical residents in LMICs.
Merali HS, Tessaro MO, Ali KQ, Morris SK, Soofi SB, Ariff S. A novel training simulator for portable ultrasound identification of incorrect newborn endotracheal tube placement–observational diagnostic accuracy study protocol. BMC Pediatrics. 2019 Dec;19:1-1 [103].	To determine whether neonatal providers that undergo a two-hour training session with a novel intubation Point Of Care Ultrasound (POCUS) simulator can then accurately detect tracheal vs. esophageal Endotracheal tube (ETTs) using POCUS, and generate a more rapid result than standard-of-care methods	This study represents the protocol for the first large investigation of the benefits of Point Of Care UltraSound (POCUS) for Endotracheal tube (ETT) confirmation in the sickest newborns undergoing intubations for respiratory support
Merchant AA, Hassan S, Baig N, Atiq H, Mahmood S, Doll A, Naseer R, Haq ZU, Shehnaz D, Haider AH, Razzak J. Methodological analysis of a community-based training initiative using the EPIS framework: an ongoing initiative to empower 10 million bystanders in CPR and bleeding control. Trauma Surgery & Acute Care Open. 2023 Nov 1;8(1):e001132 [104].	To empower 10 million bystanders with basic knowledge and skills of hands-only cardiopulmonary resuscitation (CPR) and bleeding control in a resource-limited setting by using the Exploration, Preparation, Implementation and Sustainment (EPIS) framework and to describe the feasible and sustainable application of Pakistan Life Savers Programme (PLSP) in a resource-limited setting (RLS)	The exploration phase identified a sustainable intervention to empower bystanders with basic life-saving skills. The preparation phase focused on expanding PLSP's network and attracting collaborations. The implementation phase involved piloting the intervention in diverse areas, and the sustainability phase involved integrating it into the national curriculum.
Minai F, Shafiq F, Haq MI. Value of real life (in situ) simulation training for tracheal intubation skills in medical undergraduates during short duration anesthesia rotation. Journal of Anaesthesiology Clinical Pharmacology. 2014 Oct 1;30(4):484-7 [105].	To compare the efficacy between two teaching strategies that is the real-life in situ simulation and conventional teaching as a tool for acquisition of tracheal intubation	All essential skills components of tracheal intubation in correct flow and sequence are acquired more efficiently by real life simulated training
Mirza MB, Sulaiman A, Hashmi S, Zaki S, Rehman R, Akbar R. Use of simulation based technology in pre-clinical years improves confidence and satisfaction among medical students. Journal of the Pakistan Medical Association. 2021;71(4):1296 [106].	To determine perception of medical students about learning from integrated simulated clinical skill sessions as part of the undergraduate curriculum.	Integrated clinical skills sessions improved students' interest, engagement and confidence. It should be implemented in undergraduate medical teaching curriculum.
Momin SN, Memon AS, Malik MB, Mahar PS. Surgical training in ophthalmology: Role of EyeSi in the era of simulation-based learning. JPMA. The Journal of the Pakistan Medical Association. 2022 Feb;72(2):S127-9 [107].	The aim of this study is to identify consistent or improving scores following a repetition of the same level in a cataract surgery module on EyeSi simulator.	It was found that repetitive practice on the simulator can help develop proficiency in the desired steps that can ultimately prepare the surgical trainees for real life surgery.
Mubeen K, Baig M, Abbas S, Adnan F, Lakhani A, Bhamani SS, Rehman B, Shahid S, Jan R. Helping babies breathe: assessing the effectiveness of simulation-based high-frequency recurring training in a community-based setting of Pakistan. BMC Pediatrics. 2021 Dec;21:1-0 [108].	This study aimed to evaluate the effectiveness of the Simulation-Based High-Frequency training of the Helping Babies Breathe for Community Midwives (CMW), in district Gujrat, Pakistan	The study concluded that a series of training and continuous supportive supervision and facilitation enhances Helping Babies Breathe (HBB) knowledge retention and skills
Naqvi S, Siddiqi R, Hussain SA, Batool H, Arshad H. School children training for basic life support. Journal of College of Physicians and Surgeons of Pakistan. 2011 Oct 1;21(10):611-5 [109].	To determine the background knowledge of high school children on basic life support by calculating the points scored in a multiple-choice question (MCQ) based test; to evaluate results of teaching basic life support skills to them; and assessing their power of retention by re-testing them on skills and MCQ test after the workshop.	The children showed highly significant improvement in knowledge after CPR training and retention of knowledge and skills of CPR after a 3 month period.
Nasir MU, Majid Z, Shahzad Z. Virtual reality-A means to train surgeons of tomorrow amidst Covid-19. Journal of the Pakistan Medical Association. 2022 Apr 1;72(4):797- [110].	To employ simulation-based training tools like Virtual Reality to train surgical residents in Pakistan.	The need of the hour is to employ simulation-based training tools like Virtual Reality to train surgical residents in Pakistan
Naz A, Lakhani A, Mubeen K, Amarsi Y. Experiences of community midwives receiving helping baby breathe training through the low dose high-frequency approach in Gujrat, Pakistan. Midwifery. 2022 Feb 1;105:103241 [111].	To determine the perceptions of the Helping baby breathe (HBB) trained the community midwives (CMWs) about the effectiveness of the HBB training, as well as the challenges encountered in the implementation of the HBB skills for newborn resuscitation, at their work settings.	HBB training was effective for the community midwives CMWs in terms of its usability, regarding improvement in newborn resuscitation knowledge and skills. Moreover, it enhanced confidence and satisfaction in CMWs.
Nazim SM, Riaz Q. Simulation based team training in surgery-a review. Journal of Pakistan Medical Association. 2021;71(1):S77-82 [112].	To describe the simulation based programme for team training	Simulation-Based Team Training (SBTT) includes three key elements: Didactics, which provide orientation for participants; Simulation, an event-based process involving preparatory work and role-play; and Debriefing, which occurs in three phases: reaction (identifying errors), understanding (discussing medical issues and behaviors), and summarization (offering constructive feedback).
Noorali AA, Merchant AA, Chauhan SS, Khan MA, Ehsan AN, Pervez MB, Tariq M, Fatimi S. Conceptual framework for a cardiac surgery simulation laboratory and competency-based curriculum in Pakistan-a short innovation report. Journal of Pakistan Medical Association. 2022;72(1):S103-S105 [113].	Primary Objective: The strategic establishment of a multi-tier, high fidelity cardiac surgery simulation lab, with a formal structured curriculum, would provide a platform for advanced multidisciplinary training at institutional, national, and regional levels. Secondary Objective: Further concurrent and subsequent arms include curriculum development, research in simulation education, grant acquisition and patent development.	A fully functional cardiac surgery simulation lab operates on three core principles: evidence-based medicine, comprehensive sequential training, and a tiered skill progression. It utilizes models such as a simple bench model, a virtual reality simulator, and a human performance simulator. The lab’s economic stability follows the (Thoracic Surgery Directors Association) TSDA strategy, which includes hosting an annual conference, securing grants, and developing patents.
Obaid MS, Minhas S, Effendi FN, Shaikh S, Abbas FF, Zehravi F, Tajuddin M, Ansari MB. Basic Life Support Training as Mandatory Training in Educational Institutions to Deal with Emergencies. Editorial Board.:10 [114].	To determine the immediate first aid knowledge and long term retention of its knowledge (after three months) of the students 13-18 years old participating in a volunteer program in Karachi	First aid training delivered to the volunteers was very effective and more training may be conducted at larger scales in schools.
Piryani RM, Piryani S, Shrestha U, Acharya A, Kanskar S, Shahi M, Kayastha J, Chaulagain A, Agarwal JP, Bajracharya SR. Simulation-based education workshop: perceptions of participants. Advances in Medical Education and Practice. 2019 Jul 23:547-54 [115].	The objective of this study was to evaluate perceptions of participants on simulation based education (SBE) and an SBE workshop.	Simulation-based education (SBE) workshop produced substantial differences in perceptions of participants. Participants found the workshop effective in improving knowledge and understanding of SBE.
Pirzada MA, Shaikh FA, Siddiqui NA. Role of simulation in open varicose veins surgery: a systematic review. Journal of Pakistan Medical Association. 2022;72(2):49 [116].	To assess the types and effectiveness of simulators present for open varicose vein surgery.	Reported simulation models included bench model simulator, bespoke synthetic model, inanimate synthetic model, silicone based synthetic model. Assessed domains focused on technical skills and technical ability with tools such as modified global rating scale (GRS), Objective structured assessment of technical skills (OSATS), non-technical skills (NOTECHS), imperial college evaluation of procedure-specific skill (ICEPS), task specific checklist. Overall, results showed that simulations had beneficial outcomes.
Qamar W, Qayum M, Sadiq N. Assessing the knowledge and skill of vaccination staff at Adult Vaccination Counters for COVID-19 vaccines: Simulated client method. PLOS One. 2021 Dec 23;16(12):e0261286 [117].	Determining the knowledge and skill of the vaccination staff regarding COVID-19 vaccines under which the AVCs are operating at the provincial capital of Khyber Pakhtunkhwa	Majority of the Adult Vaccination Counters (AVCs) in the provincial capital of Khyber Pakhtunkhwa didn't have a complete knowledge of COVID-19 immunization guidelines.CanSino Bio (CSB) is the least available vaccine but the vaccination staff’s knowledge on CSB was found to be perfect. Many AVCs did not know that the same brand of vaccine should be used on the second dose as on the first dose. Majority did not have a system to track the client’s first dose of vaccine. Additionally, the practice of imparting knowledge to the clients was deficient.
Qidwai W, Krishanani MK, Hashmi S, Abu Ali R. Private drug sellers education in improving prescribing practices. Journal of College of Physicians and Surgeons Pakistan. 2006;16(12):743 [118].	To determine the education of private drug sellers as an intervention tool in promoting rational use of medicines for diarrhoea at private drug outlets in a rural setting .	This study demonstrates that rational use of medicines can be promoted by providing education to private drug sellers.
Quadri KH, Rahim MF, Alam AY, Jaffery T, Zaidi Z, Iqbal M. The structure and function of a new Clinical Skills and Medical Informatics Laboratory (SCIL) in a developing country—a two year institutional experience. Journal of Pakistan Medical Association. 2008 Nov 1;58(11):612-5 [119].	To combine clinical skills and medical informatics learning by offering a combined 'SCIL' rotation to third year medical students and to determine its long-term impact.	Overall response pooled from the participating clinical groups nine months to one year after their rotation appears to be positive.
Qureshi AA, Zehra T. Simulated patient’s feedback to improve communication skills of clerkship students. BMC Medical Education. 2020 Dec;20:1-0 [120].	To explore whether simulated patients’ feedback improves the communication skills of undergraduate medical students.	Simulated participants’ feedback is key to improving medical students' communication skills. In the pretest, students scored lowest on "clarifying and summarizing" and highest on "audibility and enunciation." In the post-test, the lowest mean was for "uses clear language," while the highest was for "eye contact." Empathy was higher in the control group, likely due to more female participants.
Rafi A, Aziz W, Ather MH. Construct validity of UroSim®simulator for learning transurethral resection of bladder tumor. Turkish Journal of Urology 2020; 46(5): 373-7 [121].	To determine the construct validity of UroSim® for that we compared the performance of transurethral resection of bladder tumor (TURBT) between experts and novices.	-Statistically significant difference in mean resection time between novice and experts suggesting positive relationship between clinical experience and performance on simulator. -Safety parameters, namely, bleeding control, cuts into bladder wall, cuts into ureteric orifices, and bladder perforations were significantly different between two groups demonstrating test content of the simulator
Rahim MF, Shaikh MA, Bham SQ, Khan S, Afridi A, ul Haq S, Ansari T. Students’ perception of Simulation-based learning in Clinical Skills Lab: A One-year institutional experience at Fazaia Ruth Pfau Medical College. Pakistan Journal of Medicine and Dentistry. 2024 Feb 6;13(1):102-7 [122].	To assess the initial perceptions and experiences of medical students for the newly established clinical skills laboratory at Fazaia Ruth Pfau Medical College (FRPMC).	Majority participants reported satisfactory skills training and considered instructors’s key role in organizing and structuring the course material. Additionally, a great number of participants felt the training simulates a real hospital setting and suggested the pre-reading materials and pre-instructions [prebriefing] should be provided in advance.
Rasheed F, Bukhari F, Iqbal W, Asif M, Chaudhry HA. A low-cost unity-based virtual training simulator for laparoscopic partial nephrectomy using HTC Vive. PeerJ Computer Science. 2023 Oct 17;9:e1627 [123].	To demonstrate a low-cost virtual training simulator for laparoscopic partial nephrectomy (LPN) built on Unity and running on an HTC Vive, following goals were defined: 1) Creation of an anatomical model for the kidney with renal cell carcinoma (RCC) and minimal mesh representation. 2) Proposing position based dynamics (PBD) with the force-based approach as a stable and effective solution for soft body deformation. 3) Creating a unique LPN training experience for surgeons using a game engine. 4) Exploring the potential applications of a low-cost HTC Vive VR system for training and haptic feedback. 5) Establishing the simulator’s validity metrics and a criterion-based system for surgeon evaluation.	-All participants stated the virtual world was easy to use and that the HTC Vive haptic device helped them practice their skills. (Face validity) -Both groups agreed that the simulator cannot currently teach any experienced surgeons, but only more novices (content validity)
Rattani SA, Kurji Z, Khowaja AA, Dias JM, AliSher AN. Effectiveness of high-fidelity simulation in nursing education for end-of-life care: A quasi-experimental design. Indian Journal of Palliative Care. 2020 Jul;26(3):312 [124].	To measure the effectiveness of high-fidelity simulation to teach end of life (EOL) care in the palliative nursing course in the undergraduate nursing education program at the School of Nursing and Midwifery at Aga Khan University.	Teaching end-of-life (EOL) care through high-fidelity simulation has improved the attitudes of students towards the care of dying patients and their grieving family members. -After the simulation, a greater number of participants expressed that they would want to care for a dying person and the family -Exposure to EOL care through the simulation increased the number of participants who realized that addiction to pain medication is not a concern and that pain should be managed properly.
Raza SJ, Soomroo KQ, Ather MH. "Latex Glove" Laparoscopic pyeloplasty model: A novel method for simulated training. Urology Journal. 2011 Nov 16;8(4):283-6 [125].	To present a ‘latex glove’ laparoscopic pyeloplasty (LPP) training model and determine its construct validity for its effective use in resident training.	This ‘latex glove’ pyeloplasty model has proven its construct validity as a simulator for laparoscopic skills development.
Rehman ZU, Moosa MA, Riaz Q. Knowledge gain of the non-vascular surgeons after attending a course on traumatic vascular emergencies. Journal of the Pakistan Medical Association. 2020;70(2 (Suppl 1)):S6 [126].	To measure the effectiveness of the workshop in enhancing the knowledge and skills of the non-vascular surgeons in dealing with traumatic vascular emergencies.	-Participants found the workshop useful in improving their knowledge and skills in managing vascular emergencies. -An increase in the post-workshop scores of the participants indicates that the workshop improved their knowledge
Sabzwari SR, Afzal A, Nanji K. Mimicking rashes: Use of moulage technique in undergraduate assessment at the aga khan university, Karachi. Education for Health. 2017 Jan 1;30(1):60-3 [127].	To identify the validity, fidelity, and feasibility of the use of moulage-based simulation in summative assessment.	-The content and face validity of both dermatology stations were good, as students scored well in the OSCE stations and the stations were constructed through input of content experts (dermatologists) respectively. -Both the stations were easily developed without much cost and little time thereby proving the feasibility
Sabzwari SR, Ishaque S, Memon SJ, Musharrif SI. Creation of virtual patients for undergraduate and postgraduate medical education: An experience from Pakistan. Journal of the College of Physicians and Surgeons--Pakistan. 2023 Apr 1;33(4):457-9 [128].	To create virtual patients as an educational tool to determine their feasibility and effectiveness in clinical problem solving	-Both Virtual Patients (VPs) were easy to navigate. Majority participants reported that both the geriatric VP and pediatric VP facilitated learning through applicability to all clinical scenarios. However, few participants felt that VP was more time-consuming than traditional bedside teaching
Saeed S, Afzal A, Khalid F, Jehan F. Student experiences of simulation-based learning and its impact on their performance in objective structured clinical examination in pediatrics-A mixed method study. Pakistan Journal of Medical Sciences. 2023 Jul;39(4):978 [129].	To see the effectiveness of simulation-based integration for teaching clinical skills on student learning, their performance in their Objective Structured Clinical Exams (OCSE) exams and their experiences of learning in that environment as learners.	The simulation based medical education (SBME)) group scored significantly higher than the bedside method (BM) group on all OSCE stations, except anthropometric examination. Students valued hands-on experience in simulation-based learning and emphasized the need for motivated facilitators. Suggested improvements included smaller groups and focusing on one exam per session.
Saeed S, Hegazy NN, Malik MG, Abbas Q, Atiq H, Ali MM, Aslam A, Hashwani Y, Ahmed FB. Transforming the delivery of care from “I” to “We” by developing the crisis resource management skills in pediatric interprofessional teams to handle common emergencies through simulation. BMC Medical Education. 2024;24 [130].	To evaluate the effect of simulation-based training of interprofessional, team-based learning on acquiring crisis resource management (CRM) skills among healthcare professionals from multiple disciplines at Aga Khan University. To explore the experiences of the team regarding the training program.	Post-workshop, participants showed significant improvements in teamwork, communication, decision-making, leadership, and situational awareness. Key themes interprofessional experiences and perceptions of participants included experiential learning, practice integration, and suggestions for future sessions.
Saeed S, Khan MH, Siddiqui MM, Dhanwani A, Hussain A, Ali MM. Hybridizing video-based learning with simulation for flipping the clinical skills learning at a university hospital in Pakistan. BMC Medical Education. 2023 Aug 21;23(1):595 [131].	To measure the effectiveness of hybridizing video-based learning with simulation for the teaching of clinical skills on the perceived self-efficacy of students, the student’s satisfaction with the learning pedagogy, and their performance in their clinical exams (OSCE).	The hybridization of video-based learning with simulation significantly improved self-efficacy scores and OSCE scores in the central nervous system (CNS) and abdomen groups.The students reported that the intervention allowed them to reinforce basic concepts, retain information, and gain insight into clinical applications. The facilitator's interactive teaching and student-friendly environment contributed to the success. While video-based learning reinforced knowledge, improvements can be made to video duration and quality by increasing camera angles and checklists.
Sajid M, Jamil M, KOUSER R, Naz S, JAVEED M. Evaluation of cardiopulmonary resuscitation skills of dental students & house officers. Pakistan Oral & Dental Journal. 2017 Mar 31;37(1) [132].	To examine dentist’s medical practices of the current CPR guidelines and to recognize the precautions that should be followed to correct the deficiencies identified	Dental students dental house officers have poor level of training and knowledge on medical emergencies and cardiopulmonary resuscitation
Saleem A, Moazzam Z, Dogar SA, Qazi SH. Simulation-based training in the Paediatric Surgery population: A review of current trends and future direction. Journal of the Pakistan Medical Association. 2021;71(1 (Suppl 1)):S38 [133].	To take a look at the existing literature on the current state of simulation-based training in pediatric surgery, its potential to revolutionize pediatric surgical training, and to propose solutions to the issues that are delaying wider implementation.	Current state of simulation-based training (SBT) in pediatric surgery include: 1) Low-fidelity SBT through physical models for basic training, 2) High-fidelity SBT through computer-based simulations and virtual reality (VR) for complex tasks and progress tracking. In short, adoption of SBT is slow, though some studies show its effectiveness for medical students and surgical residents. Widespread adoption remains limited despite technological advancements. It’s constrained by the lack of validated models and high associated costs.
Saleem M, Khan Z. Healthcare Simulation: An effective way of learning in health care. Pakistan Journal of Medical Sciences. 2023 Jul;39(4):1185 [134].	The main objective of this paper was to use existing literature to explore aspects of simulation in healthcare teaching.	Simulation in medical education has advanced from basic anatomical models to sophisticated technologies like electronic patients, standardized patients, and manikins, grounded in Kolb's experiential learning theory. It’s widely used in emergency medicine, surgical residencies, and COVID-19 training. Benefits include effective teaching, skill refinement, and assessment, but challenges arise with real-world judgment, poorly designed manikins, uncertain skill transfer, high costs, and time-consuming implementation.
Salim N, Shoaib A, Amir MA, Shiraz MI, Ayaz A, Shahid AR. Impact of simulation-based training on transesophageal echocardiography learning: A systematic review and meta-analysis of randomized controlled trials. Current Problems in Cardiology. 2024 May 23:102679- [135].	To increase the statistical power of the available evidence by combining it to assess the benefits of simulator versus non simulator training for Transesophageal Echocardiography (TEE).	TEE simulator training resulted in higher skill and knowledge posttest scores and better training satisfaction, compared with non simulator training.
Saqib SU, Saleem O, Riaz A, Riaz Q, Zafar H. Impact of a global pandemic on surgical education and training-review, response, and reflection. Journal of the Pakistan Medical Association. 2021;71(1 (Suppl 1)):S49 [136].	To highlight the impact of coronavirus disease on surgical training and institutions' response to the situation in order to continue surgical training, and lessons learnt from the pandemic	Challenges for surgical training in COVID-19: Suspension of clinical activities, shortage of personal protective equipment (PPE) and surgical exposure, COVID-19 exposure/ testing/ self-isolation of trainees, cancellation of elective surgeries, senior surgeons only performing emergency surgeries, depletion of psychological health of trainees Impact on specialty based challenges: Reduced exposure of trainees in oral and maxillofacial, cardiothoracic, otorhinolaryngology, general surgery and surgical oncology Modifications and adaptations in surgical training programmes: Evidence-based use of PPE, surgical e-learning, tele-clinics, simulation platforms, virtual conferences and webinars
Sattar M, Palaniappan S, Lokman A, Shah N, Riaz Z, Khalid U. User experience design in virtual reality medical training application. Journal of the Pakistan Medical Association. 2021 Jul 1;71:1730-5 [137].	To investigate the effects of design parameters on the user experience of virtual reality medical training.	The current study reveals that VR-based learning provided a better user experience than the conventional traditional based learning (TBL) and video based learning (VBL) methodologies.
Sattar MU, Palaniappan S, Lokman A, Hassan A, Shah N, Riaz Z. Effects of virtual reality training on medical students’ learning motivation and competency. Pakistan Journal of Medical Sciences. 2019 May;35(3):852 [138].	To explore the effects of text, video and immersive technologies learning methodologies for participants’ learning in public and private medical colleges and universities of Pakistan.	The study found that virtual reality (VR) learning motivation and competency were higher than text-based learning, proving the importance of both theoretical and practical expertise in medical studies. However, text-based learning had higher mean values for learning motivation and competency, contradicting the findings that VR was the best option for medical students.
Savir S, Khan AA, Yunus RA, Rehman TA, Saeed S, Sohail M, Sharkey A, Mitchell J, Matyal R. Virtual reality: The future of invasive procedure training?. Journal of Cardiothoracic and Vascular Anesthesia. 2023 Jun 22 [139].	To present a historical review, the current status, and the potential application of VR simulation training for invasive procedures	Future potential of VR includes the provision of multisensory, almost real-world simulation to prepare trainees for the real-life environment, reducing the need for clinical experts involvement, and reusable source of training. However challenges include motion sickness 'VR sickness', cannot replicate fine movements, lack of haptic feedback
Sayyed SA, Sharkas AR, Ali Sherazi B, Dabidian A, Schwender H, Laeer S. Development and assessment of innovative high-fidelity simulation vaccination course integrating emergency cases for pharmacy undergraduates—a randomized controlled study. Vaccines. 2023 Jan 31;11(2):324 [140].	To develop an innovative training course with an HFS approach that integrates emergency handling. To investigate developing an innovative training course with an high-fidelity simulation (HFS) approach that integrates emergency handling.	Pharmacists training with high-fidelity simulation (HFS) proved to be superior to standard training in this study. Participants of the intervention group with their HFS-based training were able to perform significantly better in terms of dealing with patient information, vaccination administration, and the handling of emergency situations which is also reported by the increase in their self confidence
Shafi R, Irshad K, Iqbal M. Competency-based integrated practical examinations: Bringing relevance to basic science laboratory examinations. Medical Teacher. 2010 Oct 1;32(10):e443-7 [141].	To bring relevance to basic science laboratory practical examinations by conducting competency-based (IPEs) and to analyze its efficacy for the students	-The organization as well as clinical relevance in the practical exam was highly appreciated by the students. -Faculty found integrated practical examination (IPE) a good way of assessing application of knowledge and skills of the students
Shafiq Z, Mufti TS, Qayum I. Role of clinical skill centre in undergraduate medical education: Initial experience at Rehman Medical College Peshawar. encounter. 2017 Jan 1;2:4 [142].	To do a preliminary evaluation of the Clinical Skills Centre (CSC) training programme based on assessment of competence of students on clinical skill stations (CSS) and to measure the satisfaction level of students from the training	OSCE results revealed clinical skills center training is effective. Most of the students were satisfied with this method of teaching.
Shah N, Baig L, Shah N, Hussain RP, Shah SM. Simulation based medical education; teaching normal delivery on intermediate fidelity simulator to medical students. Journal of Pakistan Medical Association. 2017;67(10):1476 [143].	To assess the effectiveness of medium fidelity simulator in teaching normal vaginal delivery to medical students	The group trained on the simulator scored higher in the post-training test as compared to the control group. Additionally, both groups provided positive feedback with regard to the training.
Shahabuddin S, Hashmi S, Khan Y, Sami SA. Paradigm shift in the surgical training: The era of innovation, simulation and beyond. Journal of the Pakistan Medical Association. 2021;71(1 (Suppl 1)):S33 [144].	To share information about the transition of surgical training hierarchy from apprenticeship to simulation-based learning and its impact and future perspective on surgical skills training.	Surgical training has evolved from an apprenticeship model to the Halsted method and then to simulation-based approaches. The types of simulation include dry/wet-lab, minimally invasive, and robotic surgery simulators. The impact of COVID-19 led to a shift towards simulation and new methods of trainee assessment. While the future of simulation in surgical training is promising, it is limited in LMICs due to funding and curriculum gaps.
Shaikh AR, Khaliq T. Simulation based training improves laparoscopic surgical skills in trainee surgeons. Pakistan Armed Forces Medical Journal. 2021 Jan 28;71(Suppl-1):S186-92 [145].	To assess the impact of laparoscopic simulation training on surgical skills of trainee surgeons	This study demonstrated significantly improved levels of performance for all skill sets for novice surgical residents trained on LAP Simulator (LAP Sim) for minimally invasive surgical procedures.
Shams P, Ahmed I, Shahab H, Kadani Z, Khan A, Shams M, Saeed Y, Bokhari S, Khan AH. Cardiovascular fellow-in-training feedback on virtual and simulator-based learning experience during Covid-19 pandemic in a low to middle income country–A cross-sectional study. Annals of Medicine and Surgery. 2021 Sep 1;69 [146].	none- except saying, "It is imperative to analyze feedback of Cardiovascular fellow-in-training regarding this mode of learning before large scale implementation."	Healthcare professionals are shifting to virtual means of education due to COVID-19 pandemic. However, many obstacles must be dealt with to improve learning.
Siddiqui NA, Hashmi S, Naz I, Sophie Z. Validation of Task-Specific Rating Scale for Open Balloon Catheter Arterial Embolectomy: An Assessor-Blinded Quasi-Experimental Pilot Study. Annals of Vascular Diseases. 2022 Dec 25;15(4):289-94 [147].	Primary objective: To develop and validate a task-specific rating scale (TSRS) by comparing with the Global Rating Scale (GRS) for the evaluation of brachial artery embolectomy (BAE) Secondary objective: to estimate criterion cut-off points of the TSRS against overall GRS binary scores to declare trainees as successful candidates	The TSRS was found to be a valid and reliable assessment tool for BAE; however, for some domains, such as instrument handling and time and motion, it has limited reliability.
Siddiqui NA, Javed A, Pirzada A. A systematic review of simulation training for lower extremity bypass procedures. Vascular. 2023 Jul 26:17085381231192689 [148].	To determine the effectiveness of simulation training for Lower limb bypass surgery (LLBS) using Kirkpatrick Model of training evaluation	Domains assessed: Technical skills, anatomical understanding, procedural knowledge. Assessment tools: Modified Global Rating Scale (GRS), objective structured assessment of technical skills (OSATS), Imperial College Evaluation of Procedural Skill (ICEPS), self-developed checklist. Kirkpatrick (KP) level: All studies assessed at KP level 2.
Siddiqui NA, Pirzada A, Badini S, Shaikh FA. Role of simulated training for carotid endarterectomy: A systematic review. Annals of Vascular Diseases. 2022 Dec 25;15(4):253-9 [149].	To identify different types of simulators used for the training of Carotid Endarterectomy (CEA) and to assess the usefulness of all such simulators considered for simulated training on (CEA)	Five studies used plastic models, two pulsatile, with others using a bovine placental model and a 3 dimensional-printed (3DP) CEA simulator. Bovine placental model was used in one whereas 3D printed whole task CEA simulator (made of polyvinyl alcohol) was used in another study. Three studies used moderate- to high-fidelity models, while three used cadaveric or cryopreserved models. All studies video recorded all performances and used various assessment tools. Eight found simulation beneficial, one was inconclusive, and another reported no results.
Singhal N, Lockyer J, Fidler H, Keenan W, Little G, Bucher S, Qadir M, Niermeyer S. Helping Babies Breathe: global neonatal resuscitation program development and formative educational evaluation. Resuscitation. 2012 Jan 1;83(1):90-6 [150].	To develop an educational program designed to train health care providers in resource limited settings to carry out neonatal resuscitation.	A neonatal resuscitation program in resource-limited settings showed high satisfaction, self-efficacy, and knowledge gains among participants. Both facilitators and learners scored high, indicating good training and useful materials. The percentage of facilitators passing the MCQ was low pre-course but increased post-course. However, the bag and mask skill checklist performance was not only low pre-course but few facilitators and learners passed all 12 steps in the post-course.
Sohail FA, Iqbal M, Khan SP, Izhar S. Can simulation-based education replace the hands-on experience learning during the COVID-19 pandemic? International Journal of Endorsing Health Science Research. 2021; 9(3): 276-280 [151].	To highlight the importance of simulation-based education (SBE) in medical training.	No outcome reported.
Soomro KQ, Rajpar ZH, Memon II, Shah SA, Jat JA, Memon SU. Simulator based teaching of Trans-Urethral Resection of Prostate (TURP) skills in urology. IJEHSR-International Journal of Endorsing Health Science Research. 2021;9(2):211-6 [152].	To evaluate the outcome of simulation-based teaching of TURP procedural skills on virtual reality simulators and objectively assess the operative skills on the Global Rating Scale (GRS).	Simulation-based transurethral resection of the prostate (TURP) teaching significantly improves simulative operative skills in resection, bleeding control and safety parameters.
Sultana S, Nadim M, Sharif S, Khan NM, Sadia SN. Learning the pelvic examination by clerkship medical students: evaluating skills by standardized patient model: pelvic examination by clerkship medical students. Pakistan Armed Forces Medical Journal. 2015 Aug 31;65(4):548-52 [153].	To compare the effectiveness of training of pelvic examination (PE) of medical students on standardized patients (SPs) with the training on regular patients (RPs) during clinical rotations	-The students trained on standardized patients (SPs) appeared to be more skilled in their pelvic examination (PE) technique than the students who were trained on regular patients (RPs)
Tharani A, Lalani S, Mughal FB, Momin RB. Developing mental health competency in undergraduate nursing students amid pandemic: A hybrid model approach. Teaching and Learning in Nursing. 2022 Jul 1;17(3):277-81 [154].	To present the hybrid approach for teaching the clinical component of the The Mental Health Nursing (MHN) course, based on Robert Gagne’s model that was developed for the undergraduate nursing students at a private nursing institution in Pakistan	-The hybrid model of teaching was greatly appreciated by the students as it improved their learning by allowing practical application and reducing their anxiety of patient interaction. Furthermore, the feedback provided after each activity standardized the knowledge gained.
Wang MH, Dy F, Vu VK, Lim LG, Tayyab GU, Ratanachu‐ek T, Samarasekera DN, Dhir V, Jin ZD, Kida M, Seo DW. Structured endoscopic ultrasonography (EUS) training program improved knowledge and skills of trainees: Results from the Asian EUS Group. Digestive Endoscopy. 2015 Sep;27(6):687-91 [155].	To prospectively evaluate the effectiveness of a short-term structured endoscopic ultrasound (EUS) training program in improving the knowledge and skill of EUS among trainees.	Following a structured training program, trainees’ knowledge and skills in endoscopic ultrasound (EUS) improved significantly.
Waqar F, Sadia S, Khalid T. Vicarious learning during Simulations: is it more effective than hands-on training? Journal of Islamic International Medical College. 2013:14 [156].	To investigate whether the type of simulation-based learning (learning by doing versus vicarious learning) and the order in which these activities are carried out (learning by doing → vicarious learning versus vicarious learning → learning by doing) have any effect on the acquisition of knowledge on effective doctor–patient communication strategies.	Students appear to learn at least as much, if not more, about doctor–patient communication by observing their peers interact with SPs as they do from interacting with SPs themselves
Wasif M, Shah Vardag AB, Mughal A, Abbas SA, Ghaloo SK, Pasha HA. Training in temporal bone surgery: A review of current practices. Journal of Pakistan Medical Association. 2021;71(Suppl 1)(1):S99-S102 [157].	To provide an overview of the different techniques in detail that are currently being used for learning of temporal bone surgery	Current models for teaching temporal bone surgery are: 1) Cadaveric bone dissection: Pros: exposes anatomical variations, provides haptic feedback. Cons: ethical issues, requires specialized labs and infection control, single-use. 2) 3D printed models: Pros: no ethical concerns, accessible raw materials, no infection risk, customizable for anatomical differences. 3) Voxel Man simulator: Pros: provides haptic feedback, records sessions, validated simulator.
Yousuf F, Yousuf N. Agreement between simulated patients and faculty: Assessment of communication skills during objective structured clinical examination. Pakistan Journal of Medical Sciences. 2019 Nov;35(6):1570 [158].	Primary objective: To investigate the inter-rater reliability of scores between OB GYN faculty, non-OBGYN faculty and SPs for assessment of communication and counselling skills during OSCE in OB GYN. Secondary objective: to determine the reliability of scores using OB GYN faculty, non-OB GYN faculty and SP as assessors for assessment of communication and counselling skills during OSCE in OB GYN.	The SPs and non-OB GYN clinical faculty can also be used to assess communication and counseling skills on OB GYN OSCEs after required training as examiners.
Zafar A. Minimal access surgery in Pakistan: Role of virtual reality simulation in training. Isra Medical Journal. 2014;2:3 [159].	The aim of this study was to shed light on the role of virtual reality training in minimal access surgery	Various simulation-based interventional studies conducted in Pakistan report improved skills of participants after exposure to virtual reality training.
Zafar SI, Shahiryar A. Factors affecting the participants’ skills test performance in a basic life support course. Journal of College of Physicians and Surgeons Pakistan. 2019 Sep 1;29(9):810-3 [160].	To determine the effect of age, gender, varying specialties and year of residency on the practical skills of cardiopulmonary resuscitation in participants of basic life support (BLS) courses run at College of Physicians and Surgeons Pakistan (CPSP), Islamabad – Regional Centre of American Heart Association (AHA)	No significant difference was found with regard to performance between gender, age, and among various specialties; however, performance of junior residents in the BLS skills test, in comparison to senior residents, was found to be borderline insignificant .
Zafar Z, Habib H, Kols A, Assad F, Lu ER, Schuster A. Reinvigorating postpartum intrauterine contraceptive device use in Pakistan: an observational assessment of competency-based training of health providers using low-cost simulation models. BMC Medical Education. 2019 Dec;19:1-0 [161].	To assess the effectiveness and acceptability of a strategy (competency-based, blended learning approach and a portable, cost-effective training model called the Mama-U) to improve postpartum family planning in Pakistan	Competency-based training with the Mama-U model can improve the quality of postpartum intrauterine devices (PPIUD) counseling and insertion services and has the potential to extend postpartum family planning/postpartum intrauterine devices service delivery to midwives working in rural Pakistan
Zahid F, Memon A, Siddiqui M, Deewani MH, Asif O, Javer A, Khan AA. Successful use of a patient specific 3D-printed biomodel as surgical guide for excision of juvenile nasopharyngeal angiofibroma extending to skull base: A case report. Surgical Neurology International. 2024;15 [162].	To describe a case of juvenile nasopharyngeal angiofibroma utilizing a patient-specific 3D model for pre- and intra-operative planning.	Use of a 3D printed biomodel for surgical guidance allows surgeons to identify the best approach for surgery, reduce operating time, complications, recovery time and hospital stay.
Zaidi SS, Adnan U, Lewis KO, Fatima SS. Metaverse-powered basic sciences medical education: bridging the gaps for lower middle-income countries. Annals of Medicine. 2024 Dec 31;56(1):2356637 [163].	To explore the potential of the metaverse in improving basic sciences education and the limitations of lower middle-income countries (LMIC) universities. To provide fundamental learning design elements with a suggested conceptual framework for designing metaverse-based teaching to aid faculty educators and medical practitioners in understanding the key variables in immersive teaching and learning.	1.Metaverse applications: 3D human replicas, virtual labs, dissection, interdisciplinary learning, reproducible clinical scenarios. 2.Challenges: Common: Ethics, psychological effects, inclusivity. LMIC-specific: Financial issues, internet access, low acceptance. 3.Learning design: Analysis, Design, Development, Implantation, Evaluation (ADDIE), Kolb, Keller’s Attention, Relevance, Confidence, and Satisfaction (ARCS), Gagne, Successive Approximation Model (SAM), Connectivism, Situated Learning, 3D Constructivist Model.
Zain E, Talreja N, Hesarghatta Ramamurthy P, Muzaffar D, Rehman K, Khan AA, Jubapu AS, Termizi A. Assessment of quality improvement of simulation‐based learning using an evidence‐based framework in dental education. European Journal of Dental Education. 2024 Feb;28(1):358-69 [164].	to evaluate the impact of evidence-based simulation learning compared to the existing traditional-based simulation learning (TBSL) among undergraduate dental students using the Plan-Do-Study-Act (PDSA) PDSA model.	Following evidence-based simulation learning (EBSL), average knowledge scores among students were higher than those of traditional-based simulation learning groups. Additionally, the majority of the students also preferred EBSL over TBSL.
Zubair U, Zubair Z. Surgical resident training in Pakistan and benefits of simulation based training. Journal of the Pakistan Medical Association. 2020 May 1;70(5):904-8 [165].	To demonstrate the results of studies comparing the efficacy of trainees trained via the traditional apprenticeship model versus simulator-based training.	Several studies report that simulation-based teaching is superior to traditional teaching and it can also evaluate surgical skills but it needs a well built curriculum, feedback, and motivation. While few centers in Pakistan have implemented simulation-based education, additional efforts are needed to stay up with the increasing number of procedures to prevent mortality.
